# Hyaluronic Acid-PEG-Based Diels–Alder *In Situ* Forming Hydrogels for Sustained Intraocular Delivery of Bevacizumab

**DOI:** 10.1021/acs.biomac.2c00383

**Published:** 2022-06-23

**Authors:** Blessing C. Ilochonwu, Marko Mihajlovic, Roel F. Maas-Bakker, Charis Rousou, Miao Tang, Mei Chen, Wim E. Hennink, Tina Vermonden

**Affiliations:** † Department of Pharmaceutics, Utrecht Institute for Pharmaceutical Sciences, Faculty of Science, Utrecht University, PO box 80082, 3508 TB Utrecht, The Netherlands; ‡ Wellcome-Wolfson Institute for Experimental Medicine, School of Medicine, Dentistry & Biomedical Sciences, Queen’s University, Belfast BT9 7BL, U.K.

## Abstract

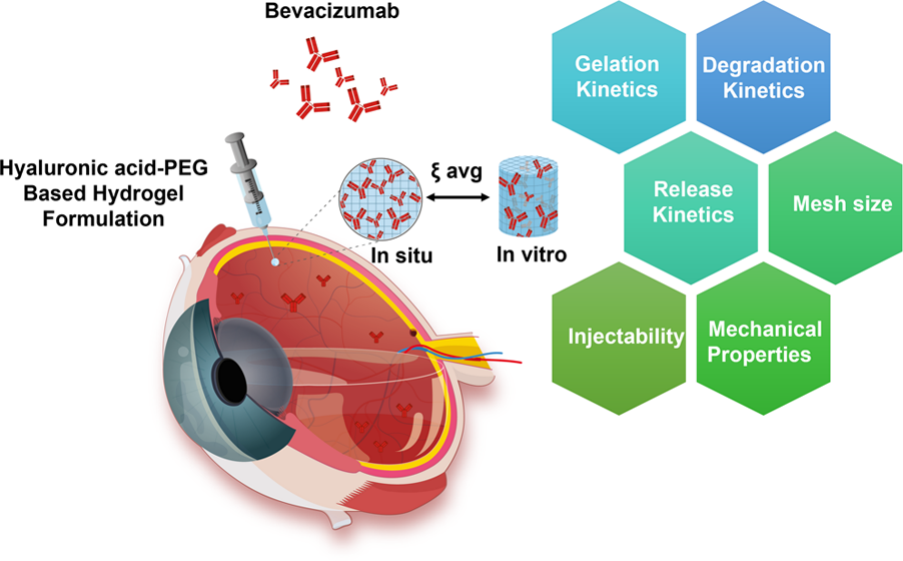

Retinal diseases are the leading cause of visual impairment worldwide. The effectiveness of antibodies for the treatment of retinal diseases has been demonstrated. Despite the clinical success, achieving sufficiently high concentrations of these protein therapeutics at the target tissue for an extended period is challenging. Patients suffering from macular degeneration often receive injections once per month. Therefore, there is a growing need for suitable systems that can help reduce the number of injections and adverse effects while improving patient complacency. This study systematically characterized degradable “*in situ*” forming hydrogels that can be easily injected into the vitreous cavity using a small needle (29G). After intravitreal injection, the formulation is designed to undergo a sol–gel phase transition at the administration site to obtain an intraocular depot system for long-term sustained release of bioactives. A Diels–Alder reaction was exploited to crosslink hyaluronic acid-bearing furan groups (HAFU) with 4 arm-PEG10K-maleimide (4APM), yielding stable hydrogels. Here, a systematic investigation of the effects of polymer composition and the ratio between functional groups on the physicochemical properties of hydrogels was performed to select the most suitable formulation for protein delivery. Rheological analysis showed rapid hydrogel formation, with the fastest gel formation within 5 min after mixing the hydrogel precursors. In this study, the mechanical properties of an *ex vivo* intravitreally formed hydrogel were investigated and compared to the *in vitro* fabricated samples. Swelling and degradation studies showed that the hydrogels are biodegradable by the retro-Diels–Alder reaction under physiological conditions. The 4APM-HAFU (ratio 1:5) hydrogel formulation showed sustained release of bevacizumab > 400 days by a combination of diffusion, swelling, and degradation. A bioassay showed that the released bevacizumab remained bioactive. The hydrogel platform described in this study offers high potential for the sustained release of therapeutic antibodies to treat ocular diseases.

## 1 Introduction

According to the World Health Organization, in 2019, approximately 2.2 billion people lived with some sort of vision impairment worldwide. Of those, 1 billion have a preventable vision impairment and 39 million are entirely blind.^1^ Ocular vascular diseases are among the leading causes of vision loss at the global level. The most prevalent ones include diabetic retinopathy (DR), diabetic macular edema (DME), and age-related macular degeneration (AMD). The number of patients suffering from these diseases is rapidly increasing in both low- and high-income countries, not only in the aging populations but also in younger individuals, representing a significant public health burden. DR is a retinal disease causing vision impairment or vision loss in diabetic patients.^2^ Over one-third of diabetic patients have signs of DR, with or without DME, making this condition one of the leading causes of visual impairment in working-age adults aged 20–71. AMD is the leading cause of irreversible blindness in elderly Europeans. Around 30–50 million people worldwide are affected by AMD, which is expected to increase in the aging population.^3^

Many studies have demonstrated that elevated levels of vascular endothelial growth factor (VEGF) play a critical role in these retinal diseases’ pathogenesis, resulting in neovascularization and vaso-permeability.^4,5^ Therefore, besides photodynamic therapy and photocoagulation, many clinical approaches aim to block VEGF signaling by delivering intravitreally injected anti-VEGF proteins.^6^ The current treatment for ocular vascular diseases includes full-length VEGF antibody (bevacizumab, Avastin), antibody fragments (ranibizumab, Lucentis), and soluble receptors (aflibercept, Eylea).^7^

Various studies have shown the effectiveness of antibodies in significantly slowing down DR and AMD progression by bolus intravitreal injections.^8,9^ This administration route’s advantage is related to rapid drug distribution to the back of the eye, increased therapeutic effect, and reduced systemic adverse events compared to other administration routes. Nevertheless, ophthalmologists consider current treatment options insufficient, as repeated injections are required to control these chronic diseases. These injections can be given at a maximum frequency of once a month because repeated intravitreal administrations result in poor patient compliance and are associated with several risks, such as bacterial endophthalmitis, retinal detachment, and hemorrhage.^10,11^ Intravitreal pharmacokinetics (PK) data show relatively rapid ocular clearance of the anti-VEGF agents (half-life around 2–14 days).^12−14^ Consequently, a high drug dose is injected into the eye and the drug concentration in the vitreous is oscillating above and below therapeutic levels in time when multiple bolus injections are administered.

Therefore, there is a growing need for suitable delivery systems to tackle the current limitations of conventional drug formulations by providing sustained release of the therapeutic agents to the back of the eye for an extended period of time, thus improving patient compliance and reducing healthcare costs.

In the past decades, tremendous efforts have been made to improve the disposition of drugs, especially bioactive proteins, in the retina using different drug delivery vehicles.^15^ Several drug delivery technologies, such as *in situ* forming hydrogels, micelles, liposomes, nanoparticles, dendrimers, microneedles, and ocular implants, are currently being investigated for ocular applications.^16−18^ However, despite these efforts, antibody-carrying implants are still currently limited on the market.^7^ Genentech’s Susvimo, previously called Port Delivery System,^19,20^ is the first and currently only FDA-approved refillable ranibizumab implant used for the treatment of neovascular age-related macular degeneration.^21^ The system allows continuous diffusion of the protein from the reservoir into the vitreous.^22^

Although this implant can significantly prolong drug release to the posterior segment of the eye, it requires invasive methods to insert the device (2.6 mm in width and 8.4 mm in length) at the target site and also to remove it. Furthermore, during phase 1 and phase 2 clinical evaluations, the occurrence of vitreous hemorrhage in a significant number of cases was noted. Although this limitation was overcome in phase 3 evaluation by modifying the surgical technique, time will tell how practical such a system will be in ocular therapy.^23^

The use of hydrogels has received increased attention as ophthalmic formulations that deliver drugs to the posterior segments. Hydrogels are three-dimensional hydrophilic polymeric networks with versatile and tunable characteristics such as biocompatibility, mechanical flexibility, tailorable release properties, and transparency.^24−28^ This type of delivery system offers several benefits for ocular drug delivery compared to the current bolus injections, including less frequent administrations, patient comfort, and potentially also cost reduction. Furthermore, hydrogels that gellify *in situ* allow entrapment of therapeutically active antibodies during network formation, facilitating local delivery and release through a minimally invasive procedure. To obtain a formulation that releases the loaded antibody for a prolonged time, its initial mobility in the gel matrix should be limited and increase in time due to swelling and degradation of the hydrogel matrix.

In this study, furan-modified hyaluronic acid (HAFU) was crosslinked with 4 arm-PEG10kDa-maleimide (4APM), yielding stable hydrogels due to Diels–Alder reaction (DA). Similar hydrogel formulations have previously been used to enable the controlled release of extracellular vesicles, and for the encapsulation and three-dimensional (3D) culture of cells in tissue engineering.^29−34^ Hyaluronic acid (HA) is a polysaccharide that is abundantly present in the vitreous of the eye.^35^ Therefore, HA has been used in vitreous substitution and to replace fluid during certain eye surgeries.^36−40^ Furthermore, HA has also been used in many ocular products designed to cleanse the eyes and offer relief from dryness in the form of eye drops.^41^ HAFU was therefore selected as a building block because of its expected compatibility with the posterior and anterior segments of the eye. Furthermore, poly(ethylene glycol) (PEG) is one of the most used polymers in drug delivery systems.^42,43^ After the first approved PEGylated products around 30 years ago,^44^ a vast amount of clinical experience has since been gained with this polymer, making it an ideal building block for hydrogels for biomedical applications. In addition, solely PEG hydrogel formulations crosslinked with DA or Micheal-type reactions have also been investigated for sustained protein release^32,33^ and potential ocular applications.^45^

Different types of crosslinking chemistry have been studied in hydrogel systems to deliver proteins to the posterior segment of the eye, as previously reviewed by Ilochonwu et al.^15^ However, slow crosslinking mechanism, permanent crosslinks, and the need for toxic catalysts and radical initiators still limit the clinical use of such systems. A major advantage of DA chemical crosslinking is that it occurs under physiological conditions avoiding the use of potentially toxic catalysts and initiators, commonly used in many existing crosslinking strategies for controlled-release hydrogel delivery systems.^46,47^ However, maleimide functional groups present in the furan-maleimide DA crosslinks can potentially react with SH and NH_2_ groups of the loaded protein,^48^ creating protein conjugates. Despite this limitation, the unique properties and advantages of DA chemistry have been gaining increasing recognition, especially when applied in biomedical applications.^46^ Although some DA-based hydrogels for ocular drug delivery have been previously studied as long-acting sustained delivery systems for bevacizumab^31,32^ with release profiles up to 100 days, there is limited information available on the influence of hydrogel composition on material’s physicochemical and structural properties and how that relates to the release profiles of therapeutic proteins.

This study aims to fill this gap by systematically examining the effects of hydrogel polymer composition and the ratio of functional groups on a series of material properties, such as gelation kinetics, injectability, mechanical properties, mesh size, degradation, and drug release kinetics for intraocular therapy. Specifically, the present work investigates a DA-crosslinked hydrogel based on HA and PEG polymers with potential application as a long-acting sustained delivery system for bevacizumab (and potentially for other anti-VEGF therapeutics). The formulation was designed and aimed to be injectable into the vitreous cavity using a small needle (29G). After injection, the aqueous polymeric solution formed a crosslinked hydrogel at the administration site, entrapping the antibody dissolved in the same solution to obtain an intraocular depot system.

Furthermore, the potential prospect of HAFU-4APM hydrogels for intraocular protein therapy was examined by testing *in situ* gel formation in porcine eye explants. Uniquely to this study, the elastic moduli (*E*) of *in vitro* and *ex vivo* formed hydrogels were determined to calculate the hydrogel mesh size. Considering the size of the used intraocular model protein, the average mesh size (ξ_avg_) of the hydrogels was designed to allow controlled release of the antibody due to a combination of swelling, diffusion, and degradation. The cytocompatibility of the formed hydrogel and its building blocks was evaluated using retinal Müller cells (QMMUC-1).

## 2 Materials and Methods

### 2.1 Materials

Lyophilized sodium hyaluronate (HA; 24 kDa) was obtained from Lifecore Biomedical (Chaska, MN). The 4-arm PEG maleimide crosslinker (4APM; 10 kDa) was purchased from JenKem Technology USA, Inc. (Beijing, China). Stock Phosphate buffered saline 10× (PBS) pH 7.4 (1.37 M NaCl, 0.027 M KCl, and 0.119 M phosphates) BioReagents were purchased from B. Braun (Melsungen, Germany). 4-(4,6-Dimethoxy-1,3,5triazin-2-yl)-4-methylmorpholiniumchloride (DMTMM) was purchased from TCI EUROPE N.V. Alexa Fluor 750 C5 maleimide dye was obtained from Thermo Fisher Scientific (Massachusetts, United States). All other commercial chemicals were purchased from Sigma-Aldrich (Zwijndrecht, the Netherlands) and used as received unless indicated otherwise. Dialysis tube membranes (molecular weight cutoff (MWCO) 10 kDa) were purchased from Fisher Scientific (Bleiswijk, the Netherlands). Avastin (100 mg/4 mL), Roche (100 mg of Bevacizumab), 240 mg of trehalose dehydrate, 4.8 mg of sodium phosphate, 1.6 mg of polysorbate 20 (Tween 20), and injection water; (pH 6.2) were kind gifts from the UMC Utrecht.

### 2.2 Functionalization of Hyaluronic Acid with Furfurylamine (HAFU)

Furan-modified HA (HAFU) derivatives were prepared by functionalizing hyaluronic acid with furfurylamine groups by means of two methods. HAFU with a low degree of substitution (DS) (30%) was synthesized by dissolving sodium hyaluronate (24 kDa; 1 g; 2.5 mmol disaccharide units) in Milli-Q-water at a concentration of 3.2 wt/v%. After dissolution, 1 mL (1.09 g; 11.2 mmol) of furfurylamine was added to the solution while stirring. The pH was adjusted with 5 M HCl to 4.75, and subsequently, 1.35 g (7.0 mol) of 1-ethyl-3-(3-dimethylaminopropyl)carbodiimide (EDC) was added. Next, 724 mg (6.0 mmol) of *N*-hydroxysuccinimide (NHS) was added while keeping the pH at 4.75. The solution was stirred at room temperature for 48 h, and the reaction was stopped by increasing the pH to 7 using 5 M NaOH. The mixture was purified by dialysis (Mw cutoff = 14 kDa) against dilute HCl (pH 3.5) containing 100–150 mM of NaCl and finally against water at 4 °C. The final product was obtained as a fluffy white powder after freeze-drying with a yield of 70–80%. To obtain HA with a higher DS (50 and 83%), HAFU was synthesized according to the procedure described by Nimmo et al.^29^ with modifications. Briefly, HA (0.40 g, 1.01 mmol disaccharide units) was dissolved in 40 mL of MES buffer (100 mM, pH 5.51) to which DMTMM was added at 6 (1.7 g, 6.0 mmol), or 2 (0.60 g, 2.0 mmol) molar ratio (relative to the −COOH groups in HA) and stirred for 10 min. Furfurylamine was subsequently added dropwise at a 2 (188.8 μL, 2.04 mmol), or 1 (90 μL, 0.97 mmol) molar ratio relative to the −COOH groups in HA. The reaction was conducted at room temperature for 24 h, and afterward, the pH was raised to 7 (using 5 M NaOH) to stop the reaction. Compared to EDC coupling, it was possible to quickly isolate the HAFU polymer produced by DMTMM coupling through precipitation in ethanol/water as the reaction byproduct remained soluble. Briefly, the products were precipitated in water/ethanol at RT with a ratio of 1:7.5 (reaction mixture H_2_O:ethanol) and washed three times with ethanol. The precipitate was vacuum-dried to obtain HAFU derivatives as a solid white powder with a yield of 84–88%. The different HAFU polymers were characterized with ^1^H NMR spectroscopy using an Agilent 400MR NMR spectrometer (Agilent Technologies, Santa Clara, CA). Data analysis was performed using MestReNova, and the chemical shifts were calibrated against the residual solvent peak (4.79 ppm for H_2_O). The ratios of the integrals of the *N*-acetyl glucosamine peak on the HA-backbone were compared with the aromatic furan peaks to determine the degree of substitution. ^1^H NMR δ (ppm): 7.5 (OCH; 1H), 6.4 (CHCH; 2H), 4.10–3.0 (protons of HA disaccharide), 2.0 (NHCOCH_3_; 3H).

### 2.3 Preparation of Hydrogels and Bevacizumab-Loaded Hydrogels

Cylindrically shaped empty HAFU-4APM hydrogels of 100 mg were prepared at 37 °C in a plastic mold (diameter 4 mm, height 5 mm). Specifically, equal amounts of HAFU and 4APM crosslinker were weighed and dissolved separately in PBS (0.13 M NaCl, 2.7 mM KCl, and 11.9 mM phosphates, pH 7.4) and mixed to obtain a total polymer concentration of 5, 10, 20 or 25 wt % unless indicated otherwise. Different molar ratios between the 4 APM crosslinker and HAFU polymers corresponding to 1:1.9; 1:3.1; 1:5.2 approximated to 1:2; 1:3; 1:5 ratios of maleimide:furan, respectively (calculated based on the DS of the functional groups present in the HA polymers) were used to prepare hydrogels with different properties. Subsequently, the samples were incubated at 37 °C for 4 h to allow crosslinking of the hydrogels.

Bevacizumab-loaded HAFU-4APM hydrogels were prepared as described above with a slight modification. HAFU polymers were dissolved in a mixture of PBS (0.13 M NaCl, 2.7 mM KCl and 11.9 mM phosphates, pH 7.4) and bevacizumab solution (50 mL; 25 mg/mL), while the 4APM crosslinker was separately dissolved in PBS. Upon dissolution, the 4APM crosslinker solution was mixed with the HAFU-bevacizumab solution and incubated at 37 °C for 4 h to enable crosslinking and protein entrapment. The HAFU-4APM hydrogels were loaded with either 1.25 or 1.50 mg of bevacizumab in the 100 mg hydrogels.

### 2.4 *In Vitro* Swelling and Degradation

Crosslinked empty hydrogels (100 mg) were prepared as described in Section 2.3 and placed in a 2 mL glass vial to perform the swelling and degradation test. The exact weight of the gel was measured (*W*_0_), after which 1 mL of PBS (pH 7.4) was added to the vial, which was subsequently incubated at 37 °C. At regular intervals, hydrogel weight was determined (*W*_t_) after removal of the PBS. Subsequently, 1 mL of fresh PBS (0.13 M NaCl, 2.7 mM KCl, and 11.9 mM phosphates, pH 7.4) was added for further incubation at 37 °C. The swelling ratio (SR) is defined as the weight at a particular time point (*W*_t_) divided by the initial hydrogel weight: SR = *W*_t_/*W*_0_.

### 2.5 Rheological Characterization

The rheological properties of the hydrogel were analyzed using a Discovery HR-2 Rheometer (TA Instruments, New Castle, DE) with a Peltier plate for temperature control. The samples were measured using a 20 mm diameter aluminum plate-plate geometry at a loading gap of 3000 μm and gap value of 200 μm. For each analysis, samples of 180 μL of different liquid hydrogel formulations were prepared as described in Section 2.3 and pipetted under the geometry on the rheometer Peltier plate. The system was covered with a solvent trap. The data were acquired at strain values within the linear viscoelastic regime (LVR). Storage (*G′*) and loss (*G″*) moduli of the different hydrogel formulations were measured during a time sweep at 37 °C with a frequency of 0.1 Hz and 1% strain. The gelation time (defined as the crossover point between *G′* and *G″*) of the different hydrogel formulations was measured at different temperatures (4, 20, and 37 °C). Hydrogel average mesh size (ξ) was calculated from the *G′* using the following equation^49−52^
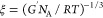
where *N*_A_ is Avogadro’s constant, *R* is the molar gas constant (8.3 J/K·mol), and *T* is the absolute temperature in K.

### 2.6 Synthesis of Dye Labeled HAFU Polymer

HAFU DS 30% (150 mg) was dissolved in 800 μL of PBS. Subsequently, 160 μL of an Alexa Fluor 750 C5 maleimide solution in dimethyl sulfoxide (DMSO, 0.5 mg/mL) was added and left to react overnight at room temperature by means of Diels–Alder reaction. Next, the solution was dialyzed against DMSO/water (1/14) for 16 h with three times solvent exchange. The product was lyophilized to obtain fluorescently labeled hyaluronic acid-furan (HAFU-750 dye) polymer as a glassy light green powder. The covalent conjugation of the dye to HA was analyzed by Shimadzu UV 2450 spectrophotometer. The HAFU-750 dye polymer (10.5 mg/mL) and the Alexa Fluor 750 C5 maleimide dye standards (0.001–0.005 mg/mL) were dissolved in 1:9 DMSO/PBS (0.13 M NaCl, 2.7 mM KCl and 11.9 mM phosphates, pH 7.4) and the absorbance UV/VIS spectra were recorded from 200 to 1000 nm with 0.5 nm resolution. Size exclusion chromatography (SEC) was used to discriminate the presence of free dye in obtained HAFU-750 dye polymer, as shown in Figure S4A.

### 2.7 *Ex Vivo* Intravitreal Injection and *In Situ* Hydrogel Formation

The enucleation of porcine eyes was performed according to the previously reported protocol by Rousou et al.^53^ Briefly, enucleation is the surgical procedure by which the entire eye is removed, including the sclera and the muscles that control eye movement are left intact. HAFU-750 dye (synthesized as described in Section 2.6)-4APM hydrogels were prepared as follows: 20 mg of HAFU-750 dye (furan DS 30%) and 20 mg of 4APM crosslinker were dissolved separately in 100 and 60 μL of PBS (0.13 M NaCl, 2.7 mM KCl and 11.9 mM phosphates, pH 7.4), respectively. Next, the hydrogel precursors were mixed to obtain a 20 wt % polymer solution (1:2 molar ratio of maleimide:furan). This solution (160 μL, 200 mg) was subsequently injected into the vitreous of an *ex vivo* porcine eye to allow *in situ* hydrogel formation. The mixture was placed in the barrel of a 1 mL insulin syringe (needle size 29G) through a pipette after removing the plunger. Before the injection, the eyes were brought at 37 °C in a water bath for 30 min. Next, the formulation was injected into the eye (vitreous body). Images of the *ex vivo* porcine eye were taken before and 5 min after intravitreal injection using an LI-COR Pearl impulse imager (LICOR, Lincoln, Nebraska) at 37 °C. The *in situ* formed hydrogel was collected from the vitreous as follows, the eyeball was held firmly with the use of a gillies forceps, and after making a small incision with a sharp blade, a spring scissor was used to cut the sclera around the cornea starting from the opening of the incision. Subsequently, the lens was removed, and the vitreous was carefully transferred into a container. The hydrogels were isolated from the vitreous body, and the mechanical properties were compared with the *in vitro* formed hydrogel (prepared in a plastic mold, Section 2.3) after 1 h incubation at 37 °C as described below.

### 2.8 *N*,*N-*Dimethylacetamide (DMA) Characterization of *In Vitro* and *Ex Vivo* Formed Hydrogels

A DMA 2980 Dynamic Mechanical Analyzer (TA Instruments, New Castle, DE) was used to determine Young’s modulus of the hydrogels. Hydrogel samples *in vitro* were prepared as described in Section 2.3, at 20 wt % (corresponding to 1:2, 1:5 molar ratios of maleimide:furan moieties in the polymers), whereas the same concentration and ratios of the *ex vivo* hydrogels were formed in vitreous, as described in Section 2.7. After extracting the hydrogels from the porcine eye vitreous body, the gels were cut to allow mechanical tests. All hydrogels (*ex vivo* and *in vitro*) were prepared with approximately 3 mm × 4 mm in height and diameter. The gels were placed between parallel plates, and a force ramp was applied at a rate of 0.5 N/min up to a total force of 8 N at room temperature. The raw data were analyzed using TA Universal Analysis software, and Young’s modulus (*E*) was calculated from the slope of the linear section (from 0 to 22% strain) of the stress–strain curve. Data are represented as mean ± standard deviation (SD) (*n* = 3 for *in vitro* gel and *n* = 6 in two porcine eyes for the *ex vivo* formed gels).

### 2.9 *In Vitro* Release from Hydrogel Network

To determine the release of bevacizumab from the different hydrogels, bevacizumab-loaded hydrogels (10, 16, or 20 wt % with molar ratio 1:2, 1:3, or 1:5 ratio of maleimide:furan) were prepared as described in Section 2.3. The release studies were performed at 37 °C, and the *in vitro* release buffer (IVR buffer) consisted of PBS (0.13 M NaCl, 2.7 mM KCl, and 11.9 mM phosphates, pH 7.4) supplemented with 0.02% NaN_3_. Bevacizumab-loaded hydrogels (100 mg) were first immersed in 500 μL of PBS. After incubation, the release samples of 200 μL were taken at predetermined time points, and 200 μL of fresh IVR buffer was added. The release samples were stored at 4 °C until analysis of protein content by size exclusion ultra-performance liquid chromatography (SE-UPLC) on an Acquity UPLC (Waters Corporation, Milford) with an FLR-detector, operated at λ_ex_ and λ_em_ of 276 and 310 nm, respectively. BEH SEC column (200 Å, 1.7 μm, 4.6 mm ×150 mm; Waters) was attached to the system and used for all measurements at room temperature. The filtered (0.2 μm) mobile phase consisted of an aqueous solution of sodium phosphate 100 mM and sodium sulfate 300 mM at pH 6.7 and was operated at a flow rate of 0.3 mL/min. Sample aliquots of 7.5 μL were injected, and the retention time of bevacizumab was 4.90 min under these conditions. The bevacizumab calibration curve’s linear range was from 7.8 μg/mL (detection limit) to 1250 μg/mL.

### 2.10 Sodium Dodecyl Sulfate Polyacryl Amide Gel Electrophoresis (SDS-PAGE)

To study possible structural modifications of the protein with hydrogel precursors, sodium dodecyl sulfate polyacryl amide gel electrophoresis (SDS-PAGE) was performed. Bevacizumab PBS (1 mL, 0.13 M NaCl, 2.7 mM KCl, and 11.9 mM phosphates, pH 7.4) solution (1 mg/mL) was incubated with 5 mg of hydrogel precursors, either HAFU (DS 30%, 83%) or 4APM for 1 h and 5 days. Bevacizumab solution (1 mg/mL) was used as a control, and Precision Plus Protein Unstained Protein Standards 10–250 kDa (Bio-Rad, Hercules, CA) were used for calibration. Possible grafting of the hydrogel polymer precursors to the antibody was studied under both reducing and nonreducing conditions. Specifically, 2 μL of samples (bevacizumab-polymer solutions or bevacizumab solution) were mixed with 7.5 μL of solution of 250 mM Tris-HCl pH 6.5; 8% SDS; 0,008% Bromophenol Blue; 40% glycerol with and without β-mercaptoethanol 5% (100 mM), and PBS was added to obtain a final volume of 30 μL. The prepared solutions were heated to 90–100 °C for 10 min. Subsequently, samples (25 μL) and standard (3 μL) were loaded into the Bolt 4–12% Bis-Tris Gel (Invitrogen Thermo Fisher Scientific, Waltham, MA) and run at 90 V for 65 min. Bolt MES (2-(*N*-morpholino)ethanesulfonic acid) was used as a running buffer. The gels were stained with Coomassie blue (Thermo Fisher, Waltham, MA) overnight and washed three times to remove excess stain. Photos were captured with the ChemiDoc (Bio-Rad, Hercules, CA).

### 2.11 Bioactivity of Released Bevacizumab by Endothelial Cell Proliferation Assay

The bioactivity of released bevacizumab was evaluated with a previously described cell proliferation assay.^54,55^ Human umbilical vein endothelial cells (HUVECs) were stimulated with 20 ng/mL VEGF. At this concentration, proliferation is maximal and enhanced approximately 6 times compared to not stimulated cells.^55^ The ability of released bevacizumab relative to that of the native protein to inhibit HUVECs VEGF-induced cell proliferation was determined. HUVECs (Lonza, Switzerland) were cultured until passage 2–5 in Endothelial Cell Basal Medium 2 (Promocell C-22211) supplemented with Endothelial Cell Growth Medium 2 Supplement Mix (Promocell C-39216). Proliferation inhibition experiments were performed in assay medium (M199 medium supplemented with 2.5% fetal bovine serum) in 96-well plates coated with rat tail collagen (Greiner Bio-One, the Netherlands).

Specifically, wells were filled with 50 μL of 25 times diluted *in vitro* release IVR sample and 50 μL of assay buffer supplemented with 80 ng/mL VEGF (final concentration 20 ng/mL) and preincubated at 37 °C and 5% CO_2_ for 1 h. Next, 4000 cells dispersed in 100 μL of assay medium were added resulting in a final volume of 200 μL per well (corresponding to 100 times IVR sample dilution). Wells without cells and filled with 200 μL of assay medium served as blank. Wells with cells stimulated with 0, 5, 10, 20, 30, 50, and 100 ng/mL (final concentrations) VEGF were included as a reference to show that a VEGF concentration of 20 ng/mL, proliferation was maximal.

Polymer solutions (3 mg/mL) of HAFU (DS 30 and 83%) and 4APM were used as controls. Cell proliferation was assessed after 92 h of incubation at 37 °C/5% CO_2_ by adding 20 μL of the Alamar Blue reagent and another 4 h of incubation.^56^ Fluorescence was measured (λ_ex_ 530 nm and λ_em_ 600 nm) with a microplate reader (Berthold Mithras LB 940, Germany). Results are expressed as relative cell proliferation, which is the proliferation normalized by the proliferation of unstimulated cells. The concentration of bioactive bevacizumab was calculated from the bevacizumab dose-dependent inhibition of VEGF stimulated HUVEC proliferation at a fixed concentration (20 ng/mL) of VEGF stimulated HUVEC proliferation.

### 2.12 Cytotoxicity on Retinal Muller Cells (QMMUC-1)

Possible cytotoxicity of hydrogels and hydrogel precursors in contact with cells was evaluated using Queen’s University Murine Müller glia Clone-1 (QMMUC-1 cells).^57^ The cells were cultured in Dulbecco’s modified eagle medium (DMEM, low glucose) (Life Technologies, Cat. No: 41965039) supplemented with 10% fetal calf serum (Life Technologies, Cat. No: 10270106) and 1% penicillin/streptomycin (PS) (Life Technologies, Cat. No: 15140122). The cells were maintained in a humidified atmosphere with 5% CO_2_ at 37 °C. HAFU (5 mg) with DS 30% and 5 mg of 4APM crosslinker were dissolved separately in 50 and 40 μL of PBS, respectively. After dissolution, the hydrogel precursors were mixed to obtain a 10 wt % hydrogel (1:2 molar ratio of maleimide:furan, respectively). The formulation mixture was transferred into a 48-well plate, partly covering the bottom of the well. After 3 h of incubation at 37 °C, the formed hydrogel adhered to the well’s bottom. Next, QMMUC-1 cells suspended in DMEM were seeded into the wells at 3000 cells/well. After 1 and 5 days of incubation, pictures of the cells were taken with a Leica DMi1 inverted microscope (Leica Microsystems, Germany) to investigate their morphology.

Alamar Blue cell viability assay was used to evaluate the effect of polymers and hydrogel leachables on QMMUC-1 cells. Hydrogels were prepared by mixing 4APM aqueous solution and HAFU (DS- 50%) aqueous solution (10 and 20 wt %) as described in Section 2.3. After crosslinking, 1 mL of PBS was added to the hydrogel and incubated at 37 °C for 9 days to extract possible soluble products that were released from the hydrogel network. Furthermore, HAFU (DS 30, 50, and 83%) and 4armPEGmalemide crosslinker were dissolved in cell culture medium to obtain different polymer concentration (0–5 and 50 mg/mL). The QUMMC-1 cells were seeded in Costar 96-well assay plates (Costar 3904, Corning, Inc., NY) at a concentration of 3000 cells/mL. After 24 h, the cell medium was removed, 20 μL of PBS solution with either possible leachables plus 100 μL of cell medium or the polymers dissolved in cell medium were added to the cells and incubated over another 24 h. After the treatment, a medium containing 1% Alamar Blue (Life Technologies, Inc., Gaithersburg, MD) was added to the cells after washing the cells twice with the same medium, and the plates were incubated at 37 °C for 2 h after which the resulting fluorescence was measured using a Glomax multi detection system (Promega, Southampton, U.K.) at 544/590 nm. Control samples containing media and Alamar Blue solution without cells were also run as an assay control. For each test material, the results were averaged from six wells at the same time (*n* = 6).

## 3 Results and Discussion

### 3.1 Hydrogel Preparation and Characterization

HA 24 kDa was chosen over higher HA molecular weights, as it is important that the final formulations have an initially low viscosity to allow injectability through a small G needle. A HAFU derivative with a low degree of substitution was prepared by the activation of carboxyl groups of HA with EDC and NHS, followed by reaction of the formed activated NHS ester with the primary amine of furfurylamine. The degree of furan substitution of HA (DS) is defined as the number of furan group residues per 100 HA disaccharide units (see equation in Supporting Information, Figure S1). ^1^H NMR analysis showed that HAFU with DS 30% was successfully obtained with this method, as shown in Figure S1. However, using this EDC/NHS method, the extent of the derivatization of HA with furfurylamine is limited due to some drawbacks, such as the necessity of accurate pH control of the reaction mixture and short half-life of EDC (∼4 h) in water at pH 5.0 compared to DMTMM, which provides superior yields, as reported by D’Este et al.^58^ Therefore, to obtain a HAFU derivative with a high DS, DMTMM was used as activation agent. By varying the molar ratios of HA(disaccharide units)/furfurylamine/DMTMM, HAFU with different DS were synthesized. Molar ratios of 1:2:6 and 1:1:2 yielded HAFU with DS of 83 and 50%, respectively, as shown by ^1^H NMR analysis (Figure S1). The conjugation of furfurylamine to the carboxylic acid on HA was further demonstrated by Fourier transform infrared (FTIR) spectroscopic analysis as shown in Figure S2. The successful grafting of the furan groups to HA is demonstrated by the increase and shift of the peaks at 741, 1652, and 1541 cm^–1^ corresponding to =C–H– bend of furan moiety, amide I stretching, and amide II bending.

**Figure 1:**
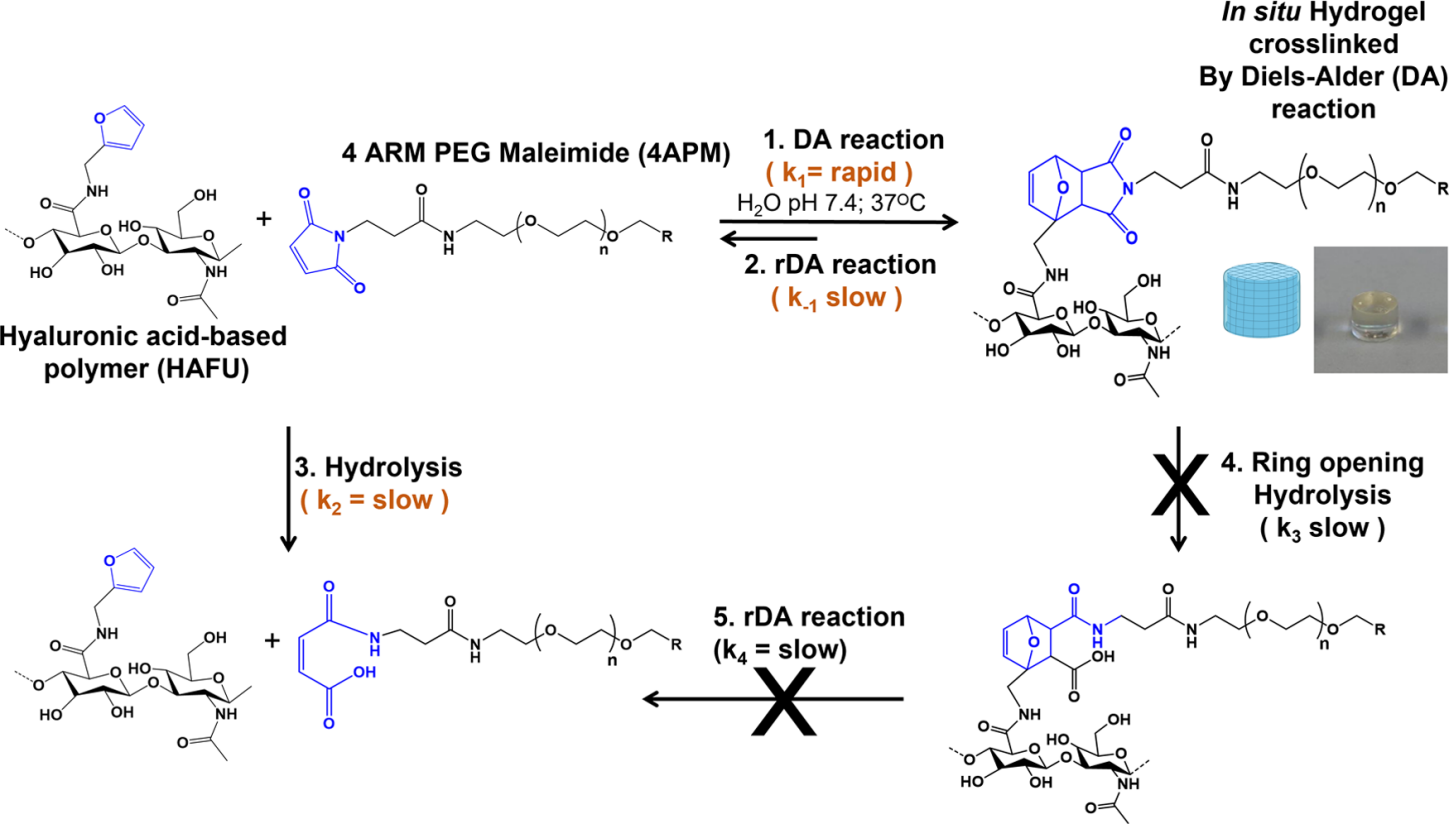
Schematic representation of hydrogel DA crosslinking reaction and potential degradation pathways. A total of six reactions are involved in a dynamic equilibrium, in which the first step is faster than the other reactions. There are five rate constants: *k*_1_, *k*_–1_, *k*_2_, *k*_3_, and *k*_4_. *k*_1_ and *k*_–1_ rate constants represent the reversible steps between reactants (HAFU and 4APM) and intermediate (DA adduct in crosslinked gel). *k*_1_ is the forward step (1 DA reaction), and *k*_–1_ is the reverse step (2 rDA reaction). *k*_2_ is the rate constant for the irreversible hydrolysis of the maleimide in the 4APM to unreactive maleamic acid. *k*_3_ and *k*_4_ describe the ring-opening rection of the DA adducts to eventually form HAFU and hydrolyzed maleamic acid in the 4APM.

Transparent (see Figure S10) and cylindrically shaped hydrogels were formed after mixing a solution of HAFU and 4APM in PBS (pH 7.4) in a plastic mold at 37 °C due to Diels–Alder (DA) reaction between the furan and the maleimide moieties^59^ (Figure 1, reaction 1). The presence of DA crosslinks in the hydrogel network was confirmed by FTIR of the dried hydrogel, as shown in Figure S3. The appearance of a new peak at 1459 cm^–1^ corresponding to the C=C bond in the Diels–Alder adduct confirms the proposed crosslinking chemistry. Hydrogels of different compositions were obtained using different polymers at varying concentrations in buffer, as shown in Table 1.

**Table 1:** Composition of the Different Hydrogels Formulation

name formulation	exact molar ratio (maleimide/furan)	polymer composition	used total polymer concentrations (wt %)
4APM-HAFU (1:2)	1:1.9	4APM/HAFU DS 30%	10–20–25%
4APM-HAFU (1:3)	1:3.1	4APM/HAFU DS 50%	5–10–15–30%
4APM-HAFU (1:5)	1:5.2	4APM/HAFU DS 83%	10–20–25%

### 3.2 Swelling and Degradation Behavior

**Figure 2:**
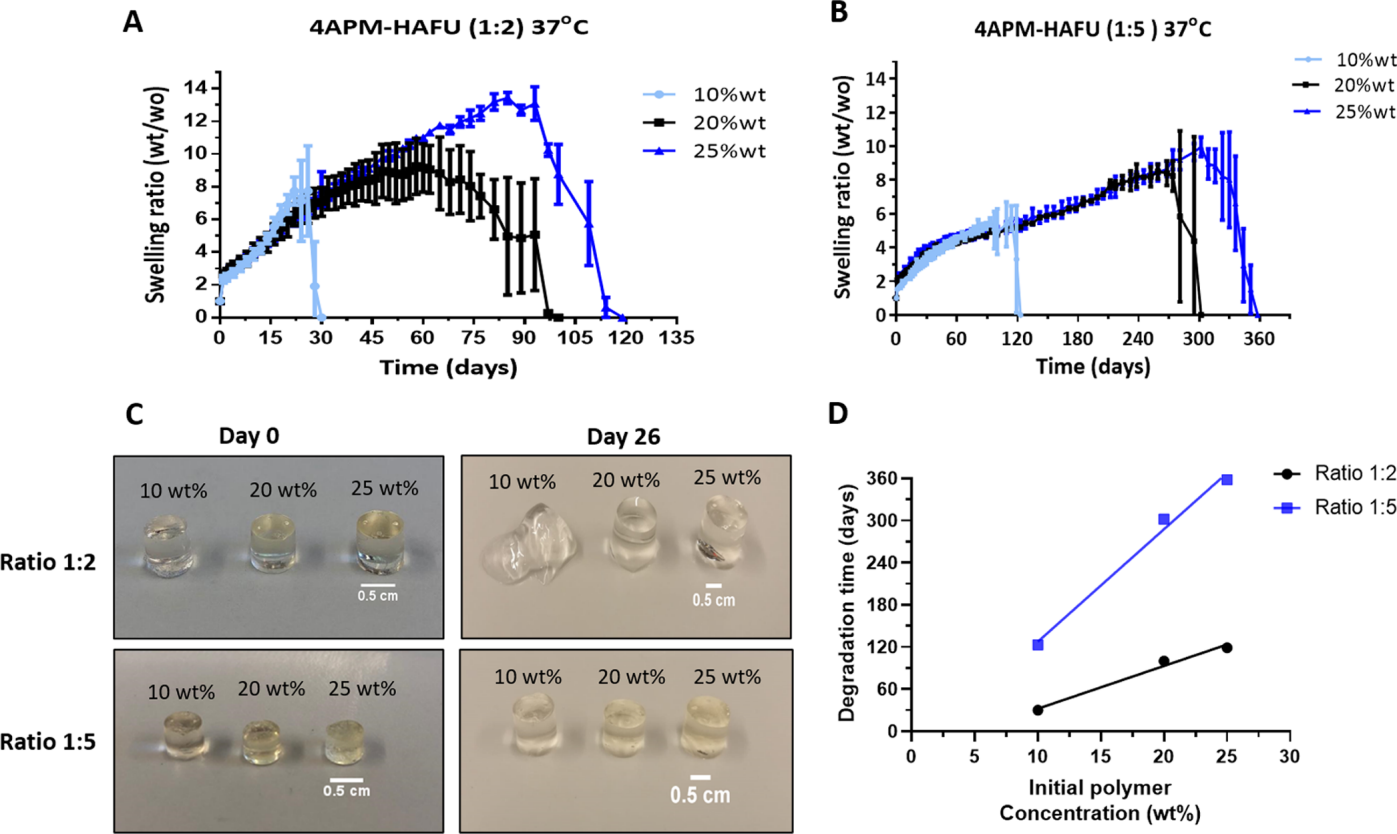
Swelling and degradation characteristics of different 4APM-HAFU DA hydrogels. (A) Swelling ratio of 10, 20, and 25 initial wt % hydrogels prepared at ratio 1:2 4APM-HAFU incubated at 37 °C in PBS pH 7.4. (B) Swelling ratio of 10, 20, and 25 initial wt % hydrogels prepared at ratio 1:5 4APM-HAFU incubated at 37 °C in PBS pH 7.4. (C) Pictures of 4APM-HAFU hydrogel (ratio 1:2 and 1:5) on day 26 of incubation at 37 °C in PBS (pH 7.4) in comparison with hydrogel after formation. (D) 4APM-HAFU (ratio 1:2 and 1:5) hydrogel degradation time as a function of the initial polymer concentration.

Hydrogel swelling and degradation properties are essential factors to evaluate when developing long-lasting hydrogels for ocular/biomedical applications. Swelling and degradation of different hydrogels during incubation in PBS (pH 7.4) and at 37 °C were measured gravimetrically. All gel formulations first absorbed water, which caused a mass increase in time up to a maximum swelling ratio followed by a gradual decrease in gel weight until they completely dissolved in the buffer (Figure 2). This increase in the gel mass and thus swelling ratio is caused by the progressive degradation of the polymer network by hydrolysis of the crosslinks by retro-Diels–Alder (rDA) reaction and subsequent water uptake (Figure 1, reaction 2). The degradation mechanism is based on the ring-opening hydrolysis of the generated maleimide after rDA to form unreactive maleamic acid (Figure 1, reaction 3), causing the removal of maleimide groups from the DA/rDA equilibrium and, therefore, consequent permanent cleavage of the crosslinks. This mechanism was confirmed by ^1^H NMR of the hydrogel degradation products (see Figure S7). The presence of the hydrolyzed (ring-opened) maleimide was identified, in line with previous reports by Kirchhof et al.^60^

Another possible pathway is caused by the direct hydrolysis of the carbonyl moiety and ring opening of the DA adduct (Figure 1, reaction 4) with subsequent rDA reaction yielding furan and unreactive maleimic acid (Figure 1, reaction 5). However, as previously reported by Kirchhof et al.,^61^ hydrogel degradation through this second pathway is not likely to occur. Gregoritza et al. and Kirchhof et al. showed that DA hydrogels based on PEG or Poloxamine can be completely degraded under physiological conditions by retro-Diels–Alder at 37 °C.^31,60,61^

Figure 2A shows that, as expected, 4APM-HAFU (molar ratio of maleimide and furan 1:2, respectively) hydrogels with 10 wt % polymer degrade faster (30 days) compared to the formulations with 20 and 25 wt % (100 and 120 days, respectively). Hydrogels with an equal overall concentration of the HAFU and 4APM building blocks, and crosslinked at a molar ratio of maleimide and furan of 1:5 showed lower swelling compared to the hydrogel crosslinked at a molar ratio of 1:2 (Figure 2A,B). Specifically, the mass of the hydrogels prepared with maleimide-furan ratio 1:5 steadily increased in weight, after which they displayed a dissolution phase which ended at 120 days (10 wt %), 300 days (20 wt %), and 360 days (25 wt %) of incubation. This suggests that with increasing furan moieties concentration, the reaction DA/rDA equilibrium shifts toward the formation of DA crosslinks (Figure 1, reaction 1), resulting in higher hydrogel stability and thus longer degradation time. The hydrogels of crosslinked 4APM-HAFU ratio 1:2 DA degraded approximately three times faster than 1:5 DA hydrogels, independent of the initial polymer concentration (Figure 2D) and all investigated hydrogels were found to be fully degradable. Images of the hydrogels after 26 days of incubation at 37 °C (Figure 2C) clearly show that the gels with a ratio of 1:2 lose their shape faster compared to gels prepared at a ratio of 1:5, due to their faster degradation kinetics.

It is important to note that hydrogel swelling and degradation behavior might differ *in vivo* due to surrounding ocular tissue, ocular clearance, and the presence of hydrolytic enzymes. In follow-up studies, hydrogel degradation and intraocular pressure should be systematically studied *in vivo* settings to rule out excessive swelling effects in the presently studied 4APM-HAFU hydrogel formulation.

### 3.3 Rheological Analysis

To be used as injectable, *in situ* forming hydrogel for intraocular drug delivery, the formulation should possess appropriate flow during injection and gelation properties, such as kinetics and stiffness, after injection. Rheological analysis was used to monitor the evolution of the storage (*G′*) and loss moduli (*G″*) as a function of time at different temperatures in relation to hydrogel compositions. A formulation composed of 15 wt % polymers (molar ratio 1:3; maleimide:furan respectively) was prepared with and without bevacizumab (1.25 mg/mL) and analyzed for gel formation using a rheometer. This formulation (15 wt % polymers (ratio 1:3)) was chosen as a model formulation with intermediate gelation kinetics to verify if there are any interactions of the protein with the hydrogel network.

**Figure 3:**
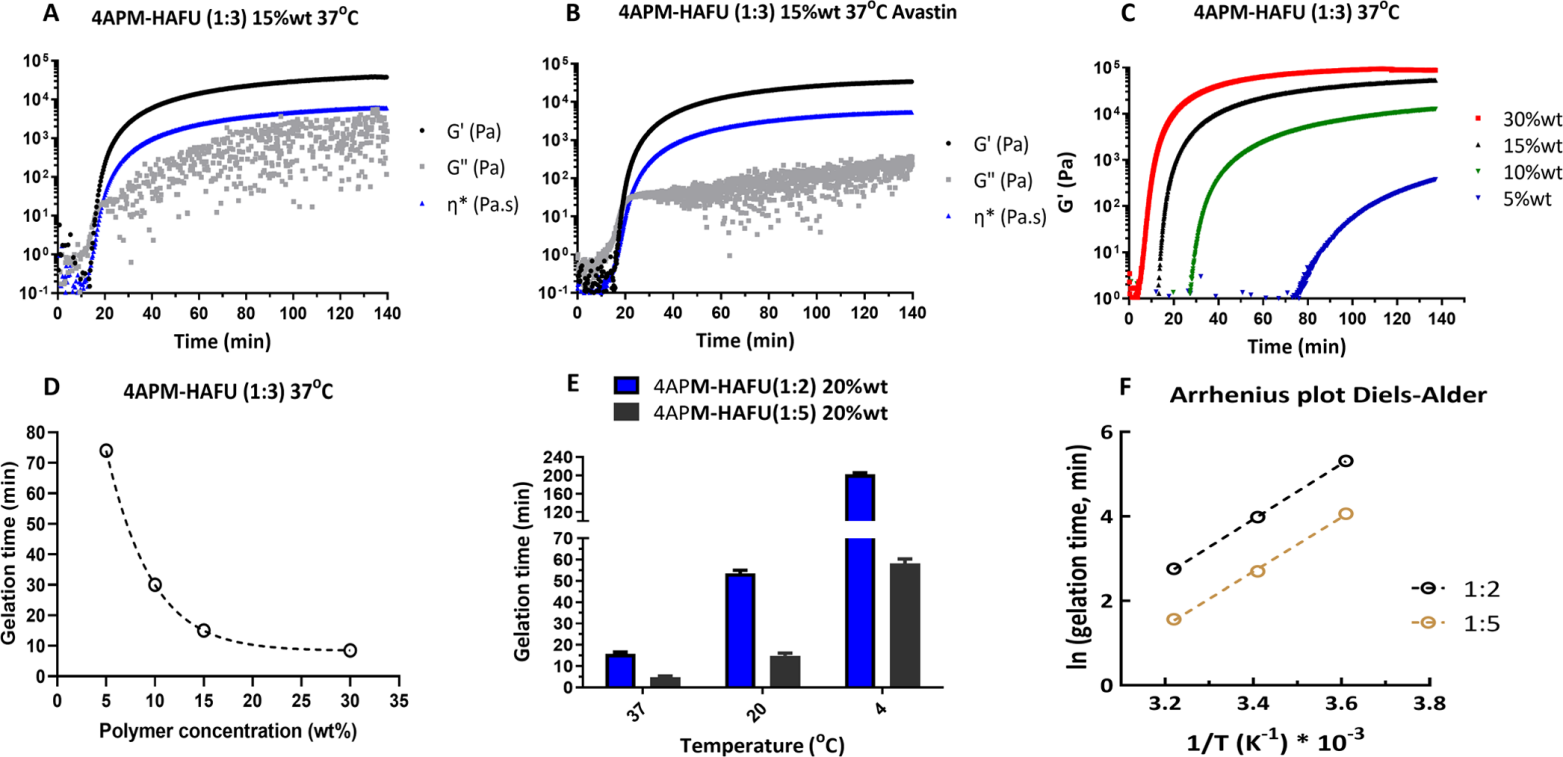
Time- and temperature-dependent rheological characteristics of 4APM-HAFU hydrogel formulation. (A, B) storage (*G′*) and loss moduli (*G″*) as a function of time of 15 wt % 4APM-HAFU (molar ratio 1:3) hydrogel formulation with and without bevacizumab. (C) *G′* as a function of time of 4APM-HAFU formulation with different polymer concentrations. (D) Gelation time of 4APM-HAFU hydrogels as a function of polymer concentration. (E) gelation time of 20 wt % 4APM-HAFU hydrogel formulations at molar ratios of 1:2 and 1:5 maleimide:furan at 37, 20, and 4 °C. (F) Arrhenius plot of the natural logarithm of the gelation time as a function of the reciprocal absolute temperature for formulations with two different maleimide:furan ratios, 1:2 (black) and 1:5 (gold). The dashed lines represent the fit of the data, and the activation energy was calculated from the slopes of the fitted lines.

The *G′*, *G″*, and complex viscosity (η*) were monitored over time, as shown in Figure 3A,B. Initially, the *G′* and *G″* were low with a complex viscosity (η*) of 0.07 Pa·s indicating a free-flowing liquid solution. Both moduli subsequently increased in time, and a crossover between *G′* and *G″* (defined here as the gelation time and corresponding to tan(δ) = 1, Figure S9) demonstrated network formation due to the reaction of the maleimide and furan functionalities. Figure 3A,B shows that the gelation time of the formulation with and without protein loading was around 17 min, indicating that the protein did not affect the gelation kinetics. Also, the final stiffness (34–38 kPa) was not affected by the presence of the protein. The *G′* increase is dependent on the polymer concentration (Figure 3C), as higher concentrations lead to faster gelling (Figure 3D) and stiffer gels. As expected, the Diels–Alder crosslinking occurred faster at higher temperatures (Figure 3E). This means that depending on the composition the formulation can be kept in the fridge (4 °C) for 1 to 3 h, before administration to a patient. In addition, formulations with a higher furan:maleimide molar ratio (5:1) resulted in faster gelation (within 5 min) at 37 °C, compared to the formulation with a 2 to 1 molar ratio of these groups (16 min). This faster gelation can be explained by the higher probability of the furan to react with the available maleimide groups. The relatively good stability upon storage at low temperature and the rapid and tailorable gelation time after intravitreal administration is a substantial advantage for possible clinical use of the formulation. The observed decrease in gelation time with an increase in temperature (from 4 to 37 °C) can be ascribed to the increased reaction rate of furan and maleimide groups.

Interestingly, it was observed that at the gelation point, 20 wt % 4APM-HAFU hydrogel formulations (molar ratio 1:2 and 1:5 maleimide:furan) had lower storage modulus with an increasing temperature (see Figure S8). This observation indicates that overall fewer crosslinks are formed at higher temperatures, which can be attributed to a slight shift in the equilibrium of forming and breaking of DA adducts.^62−65^

The temperature-dependent gelation time of the hydrogels (Figure 3F) was used to calculate the activation energy for the Diels–Alder adduct formation in the hydrogel system, as previously reported for other hydrogel-forming systems.^66,67^
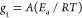
where *g*_t_ is the gelation time, *A* is the preexponential factor, *E*_a_ is the activation (kJ/mol), and *R* and *T* are the universal gas constant (8.314 J/K·mol) and the reaction temperature (in K), respectively. The calculated activation energy was 54.5 ± 1.1 and 53.4 ± 1.9 kJ/mol for 1:2 and 1:5 maleimide:furan ratios, respectively, used in the hydrogel formulations. The calculated activation energy is in agreement with previously reported values for furan-maleimide Diels–Alder systems (51.9 and 48.4 kJ/mol).^68,69^

### 3.4 *Ex Vivo* Injection and Mesh Size Determination of *In Situ* Formed Hydrogels

**Figure 4:**
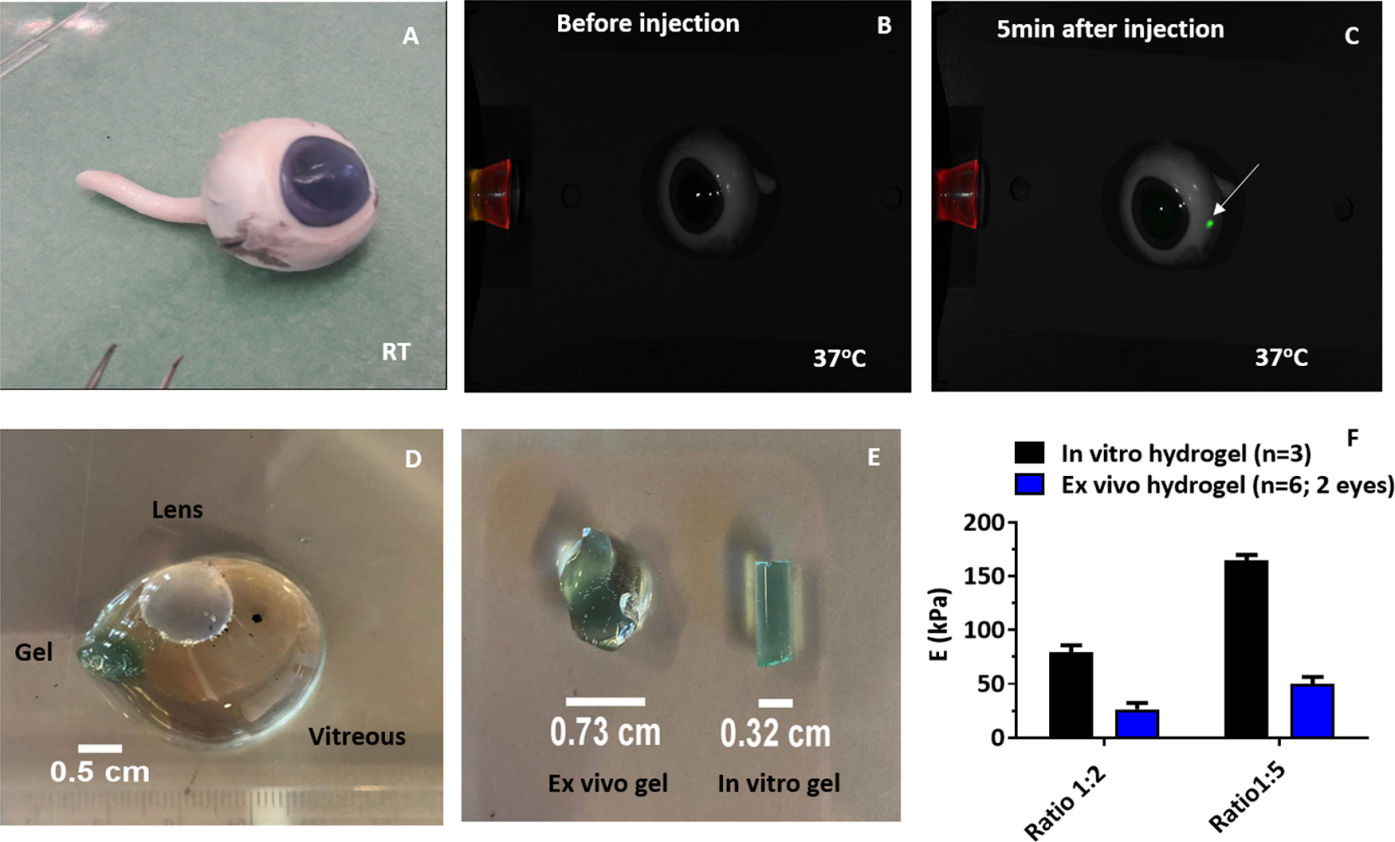
*In situ* hydrogel formation in the vitreous body after intravitreal injection. (A) Clean enucleated porcine eye at room temperature. (B, C) Fluorescent image of a porcine eye before and 5 min after intravitreal injection at 37 °C (20 wt % HAFU-750 dye/4APM ratio 1:3). (D) Vitreous image after extraction from ocular tissues, with *in situ* formed 4APM-HAFU hydrogel visible in green within the vitreous body. (E) Representative image of *ex vivo* and *in vitro* formed hydrogels. (F) *E* (kPa) of *in vitro* (*n* = 3) and *ex vivo* (*n* = 6; 2 eyes) formed 20 wt % 4APM-HAFU hydrogels at ratios 1:2 and 1:5.

To localize the formulation in the vitreous after injection, HAFU was labeled with Alexa Fluor 750 C5 maleimide by means of Diels–Alder reaction, resulting in the formation of HAFU-750 dye conjugate. The absence of free dye was demonstrated by SEC, as shown in Figure S4A. The chromatogram of the synthesized HAFU-750 dye polymer showed a UV signal at 750 nm, which corresponds to the dye fluorophore (Figure S4B). From the spectrum, it is calculated that ∼0.01% of the disaccharide units were labeled with the fluorophore showing a coupling efficiency of around 50%. The injectability of HA-PEG formulations was investigated by intravitreal injection into vitreous humor of an *ex vivo* porcine eye at 37 °C, using a 29-gauge needle. After injection (5 min), the localized presence of the formulation (in green) was observed in the vitreous at the site of injection (Figure 4C). The constrained presence within the vitreous might be due to the differences in viscosity between the vitreous body and the gel formulation. Shafaie et al.^70^ reported the complex viscosity [η*] of vitreous humor samples from the porcine eye and human eye was approximately 0.30 Pa·s at oscillatory stress of 1 Hz. The η* of 20 wt % 4APM-HAFU formulation was measured at a time sweep of 1 Hz frequency. As shown in Figure S5D, the hydrogel with a maleimide:furan ratio of 1:2 gave a complex viscosity of 0.23 ± 0.12 Pa·s, while the hydrogel with a ratio of 1:5 showed 0.75 ± 0.36 Pa·s around 3 min after mixing of the hydrogel building blocks. These values progressively increased in time due to further network formation, reaching final viscosities of 6.0 and 7.2 kPa·s, respectively Figure S5C. This relatively rapid increase in the viscosity after *in situ* crosslinking explains why after injection, the gel formulation did not significantly spread in the vitreous. Furthermore, after injection of 4APM-HAFU-750 dye hydrogel formulation (160 μL, 20 wt %) and 1 h incubation at 37 °C, the vitreous was isolated from the porcine eye to allow extraction of the formed hydrogel (in light blue), as shown in Figure 4D. The hydrogel formed *in situ* upon injection in the eye showed an irregular, bean-like shape after administration of the liquid formulation through a 29 G needle. It was found that the *ex vivo* formed hydrogel had a width of 0.73 cm and a length of 0.83 cm, whereas as a comparison, a hydrogel prepared *in vitro* took the shape of the mold (Figure 4E). Intravitreal injection of hydrogel formulations has been previously investigated in *ex vivo* and *in vivo* models.^71−73^ However, no attempts have been made to rheologically characterize *ex vivo* formed gels. Therefore, in this study, the mechanical properties of *ex vivo* intravitreally formed hydrogel were investigated for the first time. Moreover, the mesh size of the formed hydrogel was calculated and compared to that of the *in vitro* formed hydrogels. The average mesh size is an important parameter that characterizes the hydrogel network density.^74^ Therefore, the hydrogel mesh size gives information on macromolecules’ (such as therapeutic proteins) diffusivity in the gel network,^75^ a crucial aspect to consider when developing an ocular drug delivery reservoir.

To calculate the average mesh size (ξ) of *in vitro* and the isolated *ex vivo* formed hydrogels, their Young’s moduli (*E*) were determined experimentally by compression tests (Figure 4F).^76^ Furthermore, the shear modulus values (*G′*) taken at the plateau region of a time sweep curve were also experimentally determined for 20 wt % *in vitro* formed hydrogels (Figure S5A,B).

The ratio between the elastic (*E*) and shear (*G′*) moduli for the *in vitro* formed hydrogels was used to calculate the *G′* of the *ex vivo* formed hydrogel, as shown in Table 2. Specifically, it was found that for the *in vitro* hydrogels, a factor of 2.5 ± 0.2 was experimentally determined for *E* and *G′* at a frequency of 1 Hz. This value is close to the theoretical ratio, with *E* being 3 times *G′*.^77^ Considering that the *G′* of the *ex vivo* prepared hydrogels could not be experimentally determined, the same factor for the *in vitro* formed hydrogels was used. As expected, *in vitro* and *ex vivo* gels formed at molar a ratio 1:5 maleimide/furan had a higher *E* (*in vitro*: 165 ± 3 kPa; *ex vivo* 50 ± 3 kPa) and *G′* (*in vitro*: 62.4 ± 4kPa; *ex vivo* 20 kPa) compared to formulations of a molar ratio 1:2, *E* (*in vitro*: 79 ± 3.5 kPa; *ex vivo* 26 ± 2 kPa) and *G′* (*in vitro*: 32 ± 3 kPa; *ex vivo* 10.5 kPa) see Table 2. The higher *E* and *G′* values for the *in vitro* formed hydrogels (approximately 3 times higher than the *ex vivo* gels) indicate a higher crosslinking density. The obtained *G′* values were used to calculate the average mesh sizes (see equation in Section 2.5) of the different *in vitro* and *ex vivo* hydrogels by applying the rubber elasticity theory with the assumption of an affine network model, neglecting end effects of single chains, and excluding physical entanglements.^52,78^ The *ex vivo* hydrogels mesh size at a molar ratio of 1:5 (5.9 nm) was calculated to be smaller than at the ratio of 1:2 (7.4 nm), as shown in Table 2.

**Table 2:** Mesh Size of *In Vitro* and *Ex Vivo* 20 Wt % 4APM-HAFU Hydrogels as Determined by Rheological and Mechanical Measurements^a^

gel samples	*G′* (kPa)	*E* (kPa)	mesh size (nm)
*in vitro* ratio (1:2)	32 ± 3	79 ± 4	5.1
*in vitro* ratio (1:5)	62.4 ± 4	165 ± 3	3.9
*ex vivo* ratio (1:2)	b10.5	26 ± 2	7.4
*ex vivo* ratio (1:5)	b20	50 ± 3	5.9

a The *E* values of *ex vivo* formed hydrogels were determined as *n* = 6 using two porcine eyeballs, while the *E* values of the *in vitro* produced hydrogels are presented as the mean of *n* = 3 independent experiments using DMA. The *G′* value of the *in vitro* gel was obtained from the mean of the plateau region of a time sweep curve measured at a frequency of 1 Hz and a strain of 0.1%.

b Determined from the experimentally obtained *E* divided by 2.5.

The calculated mesh sizes of the *ex vivo* hydrogels are about 1.5 times greater than that of the *in vitro* formed gels, which might be because when injecting the hydrogel precursors into the eye, the polymers are diluted in the vitreous humor, which is a gelatinous tissue mainly composed of water with small amounts of hyaluronic acid, glucose, anions, cations, and collagen.^79^ The determined mesh sizes can be used to evaluate what polymer composition is suitable for releasing a therapeutic protein of a given size. The present paper reports for the first time examples of a direct comparison of average mesh size (ξ_avg_) between *in vitro* and *ex vivo* formed hydrogels. Although the *ex vivo* eye is not entirely comparable to the *in vivo* situation, it provides a valuable method for preclinical intraocular hydrogel characterization.

### 3.5 *In Vitro* Release and Structural Integrity of Bevacizumab

**Figure 5:**
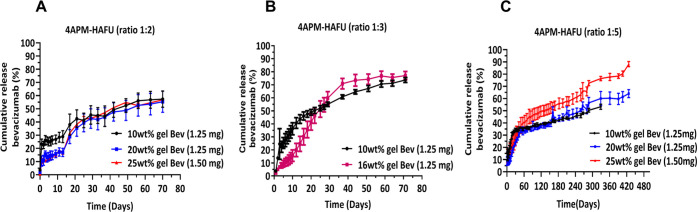
Cumulative release of bevacizumab from (A) 10, 20, 25 wt % ratio 1:2 4APM-HAFU hydrogels; (B) 10, 16 wt % ratio 1:3 4APM-HAFU hydrogels; and (C) 10, 20, 25 wt % ratio 1:5 4APM-HAFU hydrogels as determined by the SEC UPLC.

The *in vitro* release of bevacizumab from the hydrogels was studied in PBS (pH 7.4) at 37 °C. The chosen bevacizumab dose (1.25 and 1.5 mg) corresponds to the typical amount administered in clinics by bolus injection of 50 μL of Avastin (1.25 mg of bevacizumab).^80,81^Figure 5 shows that the release of bevacizumab from the 4APM-HAFU hydrogels is generally speaking dependent on the hydrogel composition, and sustained release was observed for all investigated hydrogel formulations. The release of bevacizumab from 4APM-HAFU hydrogels prepared with molar ratios of maleimide/furan 1:2 and 1:3 lasted for 70 days, after which no protein could be detected in the release samples. Specifically, hydrogels prepared at a ratio of 1:2 maleimide/furan released approximately 55% of the incorporated bevacizumab during 70 days. In the first 13 days, the 10 wt % gel released ∼29% of loaded protein while 20 and 25 wt % released ∼17% of loaded protein also during 13 days, after which the release profile was independent of the initial polymer weight fraction of the hydrogels. This observation suggests that in the first 13 days, the mesh size of the 10 wt % gel was larger than the protein size, and therefore, faster release was observed from this network compared to the 20 and 25 wt % gels. After 13 days, the mesh sizes of both the low- and high-concentration gels are larger than the protein diameter due to significant swelling of the hydrogels. No significant differences in release rate were observed after day 13 as the free volume fraction, which determines the release of proteins from hydrogels when the mesh size is greater than the protein hydrodynamic diameter^82^ as well as the gel geometries after swelling is not very different for these gels. From these results, it can be concluded that although the gelation time and the degradation kinetics largely depend on the initial polymer concentration for the gels formed based on a 1:2 maleimide/furan ratio, the release kinetics are hardly affected by these parameters.

On the other hand, 4APM-HAFU hydrogels prepared at a molar ratio of 1:3 maleimide/furan showed a two-phase release profile. The 10% gel released approximately 46% of the loaded protein in ∼16 days in an almost linear way, followed by a slower release of up to 74% of the loaded protein during the next ∼54 days. The 16 wt % 4APM-HAFU hydrogel (ratio 1:3 maleimide/furan) released ∼60% of the loaded protein within the first 30 days, followed by slower release kinetics of up to 77% of loaded bevacizumab to 70 days. Interestingly, bevacizumab was released from 4APM-HAFU hydrogels prepared at a molar ratio of 1:5 for more than 329 days, also with a two-phase release kinetics. In the first phase, 34% of the loaded protein was released from the 10 wt % gel during 30 days and from the 20 wt % gel during 50 days, while 40% was released from 25 wt % gel during 60 days. This was followed by the second phase of slower protein release. After 329 days, approximately 53% of bevacizumab was released from the 10 wt % gel, while 64% was released from the 20 wt % gel after 427 days. Remarkably, the 25 wt % gel prepared at a ratio of 1:5 4APM-HAFU showed nearly complete bevacizumab release over the measured time frame of 427 days. For comparison, previously Kirchhof et al.^61^ Gregoritza et al.^31^ reported up to ∼100 days of sustained bevacizumab release from DA-based hydrogels.

As reported in Table 2, the *in vitro* and *ex vivo* formed hydrogels’ mesh size is between 3.9 and 7.4 nm, and these values increase during hydrogel swelling and degradation. Therefore, it is expected and also in agreement with the *in vitro* data, that the release of a monoclonal antibody such as bevacizumab with a hydrodynamic radius of around 6.5 nm^83^ is likely controlled by a combination of swelling, degradation, and diffusion and subsequently multiple phase release profiles can be observed. However, for the *ex vivo* formed 4APM-HAFU (molar ratio 1:2) hydrogel, the calculated average ξ_avg_ is around 7.4 nm (Table 2), which means that after 2 times swelling the loaded protein will be released mainly by diffusion from regions with mesh size above the size of the protein while part of the loaded protein molecules might be entrapped in the hydrogel in regions with mesh sizes <2 × 6.52 nm and can therefore only be released upon swelling and degradation of the hydrogel.

In general, other proteins administered through intravitreal injections do not exceed a hydrodynamic radius of 10 nm, e.g., aflibercept (5.20 nm); ranibizumab (4.1 nm).^84,85^ Therefore, knowing the difference in hydrogel initial mesh size *in vitro* (5.1–3.9 nm) and *ex vivo* (7.4–5.9 nm), protein release kinetics could potentially be predicted based on their diameter. Nevertheless, it is essential to note that protein-network interactions might also affect the drug release rate.

**Figure 6:**
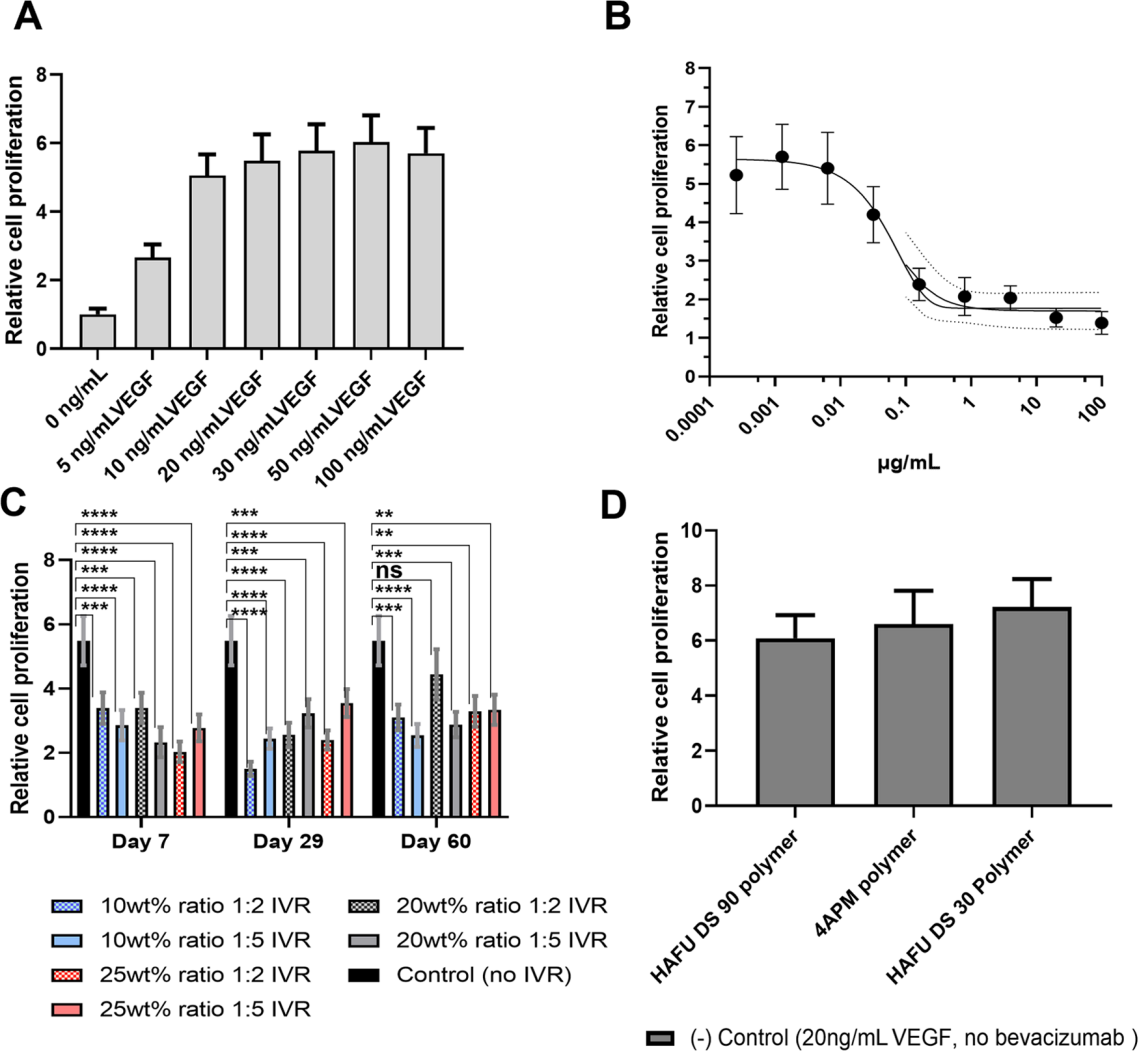
Bioactivity of released bevacizumab from 4APM-HAFU hydrogel networks. (A) Relative cell proliferation of HUVECs stimulated by VEGF (0–100 ng/mL). (B) Bevacizumab dose-dependent inhibition of VEGF (20 ng/mL.) stimulated HUVEC proliferation. (C) Bioactivity of 100 times diluted IVR samples (on days 7, 29, 60) on cell proliferation of HUVECs treated with 20 ng/mL VEGF; * *p* < 0.05, ** *p* < 0.01, *** *p* < 0.001, **** *p* < 0.0001 and ns for *p* > 0.05 compared to control treated with 20 ng/mL VEGF (One-way analysis of variance (ANOVA), multiple comparison test). (D) Effect of the polymer precursors on proliferation (cells were treated with 20 ng/mL VEGF). Data are presented as mean ± SD of three independent.

The release curves of Figure 5 span a much longer time frame compared to the degradation curves in Figure 2 for the empty hydrogels. Noteworthy, the protein-loaded hydrogels were visually discernable in the release medium over the complete release period. This observation means that the protein-loaded hydrogels degrade much slower compared to the empty hydrogels. Specifically, we observed a factor of 2–3 slower degradation rate for the protein-loaded 4APM-HAFU hydrogels compared to the corresponding empty hydrogels. The retarded degradation of the protein-loaded gels may have a number of reasons. First, electrostatic interactions between the negatively charged hydrogel network and the slightly cationic bevacizumab (isoelectric point 8.3) may play a role in retaining the protein in the gel.^86^ Second, the encapsulated protein can act as a chemical crosslinker when one protein molecule reacts with two or more maleimide groups present in the polymer network. Amine and thiol residues of proteins can react with maleimides by a Michael-type addition.^87^ For bevacizumab, reactivity with amines is more likely since thiol functionalities are disulfide bridged in this protein.^88^ Free maleimide moieties available during the formation of Diels–Alder crosslinks can potentially react with the protein both during and after hydrogel formation. These grafted proteins can only be released upon the network’s degradation. The occurrence of these grafting reactions, leading to protein-polymer conjugates, were indeed confirmed by SDS-PAGE analysis under reducing and nonreducing conditions. Incubation of bevacizumab for 1 h with 4APM polymer resulted in the coupling of around one PEG chain after 1 h, and more extensive modification was observed after five days of incubation, and approximately, on average, three PEG chains were coupled to the protein, as shown in SI-Figure 6A. As expected, the HAFU DS30 and 83 did not react with the protein even after 5 days of incubation at 37 °C, justifying the choice to dissolve HAFU in the protein solution.

When preparing a hydrogel at a molar ratio of maleimide and furan groups of 1:1, in principle, 100% conversion of both reactants is possible. However, crosslinks are formed randomly, and the mismatched reactive groups can result in the presence of free reactive maleimide groups in the polymer network.^89^ For this reason, bevacizumab was loaded in hydrogel formulations containing higher ratios of furan functional groups compared to maleimide groups to minimize the presence of the latter, thus limiting protein modification. The excess furan was also chosen to minimize possible side reactions of the maleimide with biological systems as furan is considered to be safer. During the release study, a significant extent of modification was still seen for the protein released after 21 days from the 1:2 4APM-HAFU hydrogels. Nevertheless, when proteins are linked to the hydrogels, the quantitative release of modified protein from a hydrogel matrix can still occur upon complete degradation of the network. However, complete release was not observed in this study, likely because large soluble conjugates composed of multiple PEG chains and protein molecules linked together are formed, which are captured by the precolumn in the analysis method, and consequently, they are not detected. Importantly, the extent of modification was substantially reduced using a higher concentration of furan polymer than maleimide polymer in a molar ratio of 5:1, respectively (Figure S6B). In this hydrogel, the concentration of free maleimide groups is low and thus unwanted reaction with bevacizumab is minimized and prolonged-release of approximately 90% native protein after 3 months was achieved. Figure 5C shows that by increasing the furan/maleimide molar ratios of the formulations, a nearly complete release of protein was achieved (88% for 25 wt % 4APM-HAFU molar ratio of 1:5). Overall, in agreement with the swelling and degradation study, the initial polymer concentration has a relatively small effect on the release rate compared to the furan/maleimide ratio.

The bioactivity of released bevacizumab was analyzed by a cell proliferation assay as shown in Figure 6. The bioactivity of the released protein from 4APM-HAFU hydrogels (ratio 1:2 and 1:5) after days 7, 29, and 60 was studied. The bioactivity of bevacizumab after longer release time points was not measured as time-related unspecific effects, such as oxidation, deamidation, aggregation, and adsorption to the vial surfaces are likely to occur.

Figure 6A shows that the relative cell proliferation increases approximately 6 times compared to nontreated HUVECs with VEGF concentration up to 20 ng/mL and levels off at higher concentrations. Therefore, the inhibitory effect of bevacizumab was investigated for cells in the presence of 20 ng/mL VEGF and a sigmoidal dose–response curve was observed (Figure 6B).

As shown in Figure 6C, after dilution of the *in vitro* release (IVR) samples 100 times to reach concentrations within the descending range of the dose-dependent inhibition curve (0.1–0.01 μg/mL), proliferation of HUVECs was measured. All groups showed a significant reduction (*p* < 0.05) of cell proliferation compared to the maximal cell proliferation (Figure 6C), except for the formulation 20 wt % ratio 1:2 IVR day 60, probably due to dilution close to the detection limit. This reduction can be explained by the inactivation of VEGF due to bevacizumab in the release samples and as expected polymer precursors (which can be present in the IVR) did not inactivate VEGF Figure 6D. Quantitative correlation between the released bevacizumab concentration (detected by SEC) and reduction in HUVEC proliferation cannot be directly obtained due to the relatively high inaccuracy of the biological assay. Nevertheless, after 60 days, the released bevacizumab from the hydrogel formulations was still able to reduce the activity of VEGF stimulated proliferative HUVECs indicating that the released proteins were still active.

### 3.6 Cytocompatibility Studies

**Figure 7:**
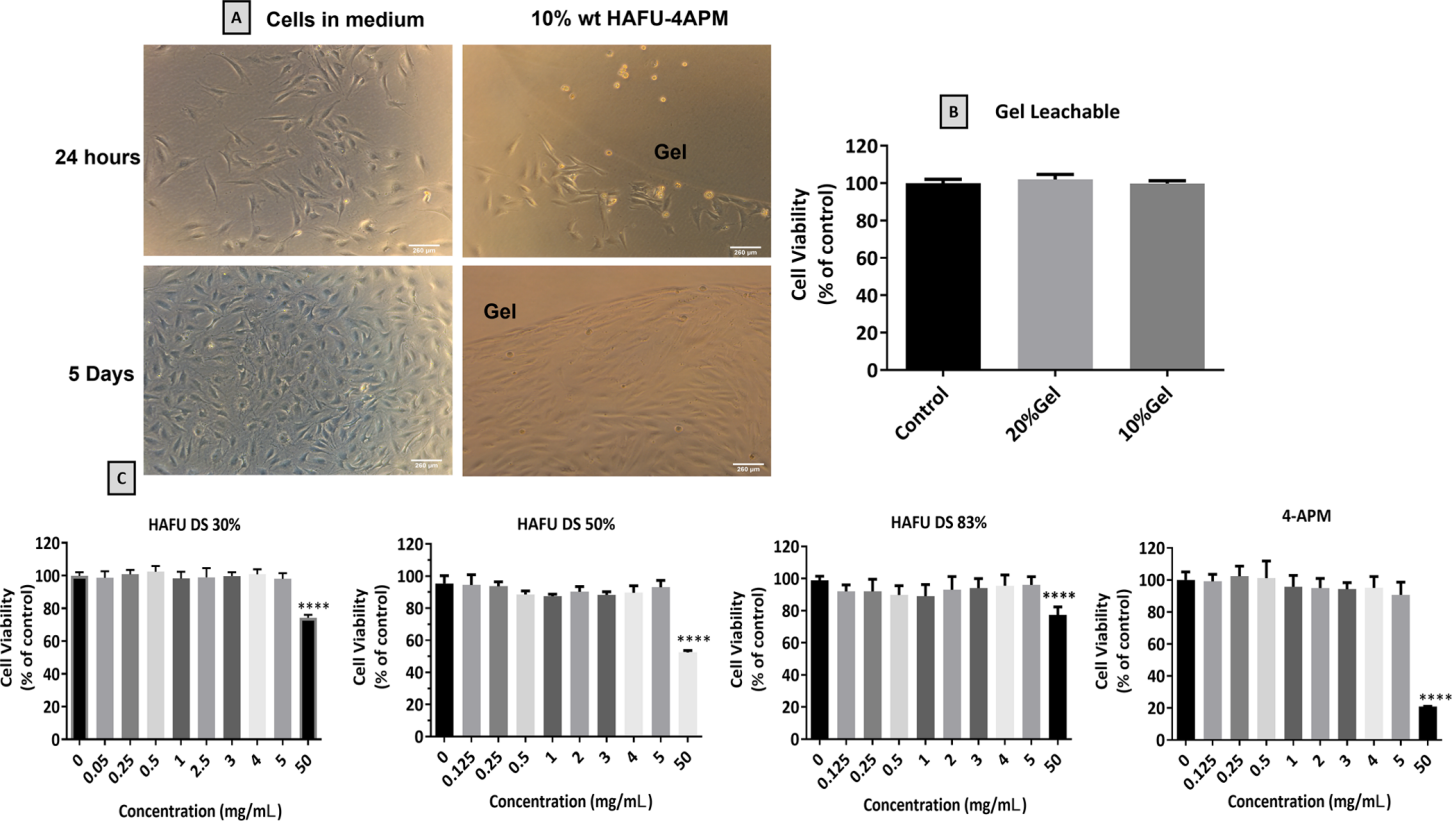
Cytocompatibility of hydrogel and hydrogel precursors on QMMUC-1 cells. (A) Morphology of QMMUC-1 cell after 24 h and 5 days co-incubated in direct contact with 10 wt % 4APM-HAFU hydrogel; the location of the hydrogels and the scale bar (260 μm) are indicated in the images. (B) QMMUC-1 cell viability in the presence of hydrogel leachables from 10 and 20 wt % 4APM-HAFU hydrogels after 24 h incubation. (C) Cell viability (Alamar Blue) of QMMUC-1 cells after 24 h incubation with polymers (HAFU DS 30, 50, 83% and 4APM) across a concentration range of 0.125–50 mg/mL. **** *p* < 0.0001 compared to control untreated cells (one-way ANOVA, multiple comparison test), (*n* = 6).

Possible cytotoxicity of the hydrogel polymer precursors and hydrogel on contacting cells was evaluated using the QMMUC-1 cell line. Müller glial is a primary retinal glial cell type and contributes to maintaining retinal structure and homeostasis.^57^ Furthermore, Müller glia are known to play a role in the pathogenesis of diabetic retinopathy and hypoxia retinal vascular disorders as they produce VEGF, which plays an important role in retinal inflammation and vascular leakage in diabetic retinopathy.^90^ Clearly, damage to these cells will drastically disrupt normal retinal function. Figure 7A shows that the QMMUC-1 cells surrounding the gels adhered to, spread, and grew on the cell culture dish with normal morphology and proliferation after 24 h and 5 days of culture. This observation indicates that the hydrogels do not release toxic leachables for these cells. QMMUC-1 cells located on top of the hydrogel’s surface did not spread and grow, and they were found floating with different morphology compared to the control. This poor cell adhesion on the surface of the hydrogel might be due to the presence of a 4-arm PEG crosslinker in the hydrogel network, as also discussed by Yu et al.^45^ In another study, Nimmo et al.^29^ showed that MDA-MB-231 cells (human breast cancer cell line) placed on top of HA-PEG hydrogels remained round for the first 24 h, after which the cells began to adopt a flattened morphology, suggesting cell attachment. The authors discussed this was due to the expression of CD44 cell surface antigen, a receptor for HA,^91^ allowing for cell interaction and potential adhesion to the gel surface; however, a significant number of cells did not adhere to the gels and were removed during media exchange. Nevertheless, although CD44 cell surface antigen is expressed on mature Müller glial cells,^92^ the cells did not attach on top of the gel surface but were found in direct contact with the gel after the 5 days of study. It is essential to note that intravitreally implanted hydrogels are not required to have cell adhesion properties when used as drug reservoirs, as they are developed to have limited interaction with cellular tissue surrounding the vitreous environment.

Alamar Blue cell viability assay was used to evaluate the effect of hydrogel leachables and polymer precursors on QMMUC-1 cells. Figure 7B shows that hydrogel leachables (from 10 and 20 wt % gel) did not affect cell viability (approximately 100% of the cells were metabolically active after 24 h exposure). (This result is in accordance with the direct contact experiments.)

As discussed in Section 3.2, the HA-PEG hydrogels studied are fully degradable by retro-Diels–Alder reaction. This means that the hydrogel precursors could gradually detach from the network and diffuse in the vitreous with potential toxicity to the surrounding tissues. Therefore, different polymer concentrations of 4APM crosslinker and HAFU DS 30, 50, 83% (0.125–50 mg/mL) were used to evaluate cytocompatibility with QMMUC-1 cells. From the results shown in Figure 7C, it is clear that the polymers were well tolerated by the cells up to the concentration of 5 mg/mL. However, at a polymer concentration of 50 mg/mL, cell viability was significantly (*p* < 0.0001) reduced to 75, 59, and 77% for HAFU polymer with DS of 30, 50, and 83%, respectively, while more pronounced toxicity was observed for the 4APM crosslinker, reducing the viability to 21%. Considering that the volume of the vitreous humor in the adult human eye is approximately 4 mL,^93^ it is expected that after intravitreal injection of 50 μL of PEG-HA hydrogel polymer precursors, the concentration of the individual components in the eye would be maximally between 0.625 and 1.25 mg/mL, which is shown to be well tolerated by the QMMUC-1 cells.

## 4 Conclusions

In this study, an intravitreal *in situ* forming DA-crosslinked hydrogel based on HA and PEG polymers with potential application as a long-acting sustained delivery system for bevacizumab and potentially for other anti-VEGF therapeutics was investigated. The prospect of the system for treating retinal diseases was examined step by step by testing hydrogel gelation kinetics, mechanical properties, injectability, biodegradability, sustained release of bevacizumab, and cytocompatibility to retinal cells. In summary, we showed that gelation time and hydrogel final stiffness are strongly dependent on temperature and ratios of the reacting furan and maleimide groups present on HA and PEG, respectively. The obtained hydrogels were fully degradable under physiological conditions due to the retro-Diels–Alder reaction. Formulations could be easily injected into the vitreous body of an *ex vivo* porcine eye through a 29G needle, and crosslinked hydrogels were obtained, whose mesh size was determined by mechanical analysis. To the best of our knowledge, the reported method was the first example of a direct comparison of hydrogel mesh size *in vitro* and *ex vivo*, providing a valuable tool for preclinical intraocular hydrogel characterization. The hydrogels showed no toxicity to QMMUC-1 at the used concentrations *in vitro*. Concluding, 4APM-HAFU hydrogels formed at a maleimide/furan molar ratio of 1:3 provide sustained release of bevacizumab for 2 months. This formulation can therefore potentially be used for therapy to replace the monthly injection by an injection every 2 months. For prolonging chronic therapy, the hydrogel formulation with a maleimide/furan molar ratio of 1:5 could be considered as this formulation showed sustained release of bevacizumab for up to a year. However, further research on whether indeed bioactive protein is released during this time frame is needed.

## Supporting Information Available

^1^H NMR spectra of HAFU DS 30, 50, 83%; FTIR spectra of HA, HAFU DS 30% and HAFU 83%; FTIR spectra of 4APM, HAFU DS 83%, and HAFU-4APM hydrogel; SEC chromatograms and UV–vis spectra of HAFU-750 dye polymer; rheological and mechanical characterization of 4APM-HAFU hydrogel; SDS-page of bevacizumab and SEC chromatograms of 10–20 wt % 4APM-HAFU hydrogels after 21–63 days of release; ^1^H NMR spectra hydrogel degradation product; storage modulus (Pa) at gelation time of 4APM-HAFU hydrogels; storage (*G′*), loss moduli (*G″*), and tan (δ) in time; and image of transparent HAFU-4APM hydrogels (PDF)
