# Supplementary material for: Hyaluronic Acid-PEG-Based Diels–Alder In Situ Forming Hydrogels for Sustained Intraocular Delivery of Bevacizumab
Source: Biomacromolecules. 2022 Jun 23;23(7):2914–29. doi: 10.1021/acs.biomac.2c00383 (PMC9277588; doi:10.1021/acs.biomac.2c00383)
Supplement: Supplementary file 1 — bm2c00383_si_001.pdf [file bm2c00383_si_001.pdf]

Supporting information:

HYALURONIC ACID-PEG BASED DIELS-ALDER *IN SITU* FORMING HYDROGELS FOR SUSTAINED INTRAOCULAR DELIVERY OF BEVACIZUMAB

Blessing C. Ilochonwu[1], Marko Mihajlovic[1], Roel F. Maas-Bakker[1], Charis Rousou[1], Miao Tang[2], Mei Chen[2], Wim E. Hennink[1], Tina Vermonden[1*]

[*]Corresponding author, E-mail: t.vermonden@uu.nl

[1]*Department of Pharmaceutics, Utrecht Institute for Pharmaceutical Sciences, Faculty of Science, Utrecht University, PO box 80082, 3508 TB Utrecht, the Netherlands.*

[2]*Wellcome-Wolfson Institute for Experimental Medicine, School of Medicine, Dentistry & Biomedical Sciences, Queen's University Belfast, UK.*

In Figure S1, the presence of furan protons on the HA-backbone are shown by the aromatic peak at 6.4 (CHCH, 2 protons) and 7.5 (OCH, 1 proton) ppm. The degree of substitution (DS %) was determined by ^{1}H-NMR using the following equation:

$$\mathbf{DS\ (\%)=\frac{I_{7.5}/1}{I_{2.0}/3}\ x\ 100}$$

Where $I_{7.5}$ is the integral at 7.5ppm and $I_{2.0}$ integral at 2ppm of N-acetyl glucosamine ($NHCOCH_3$) in native HA.

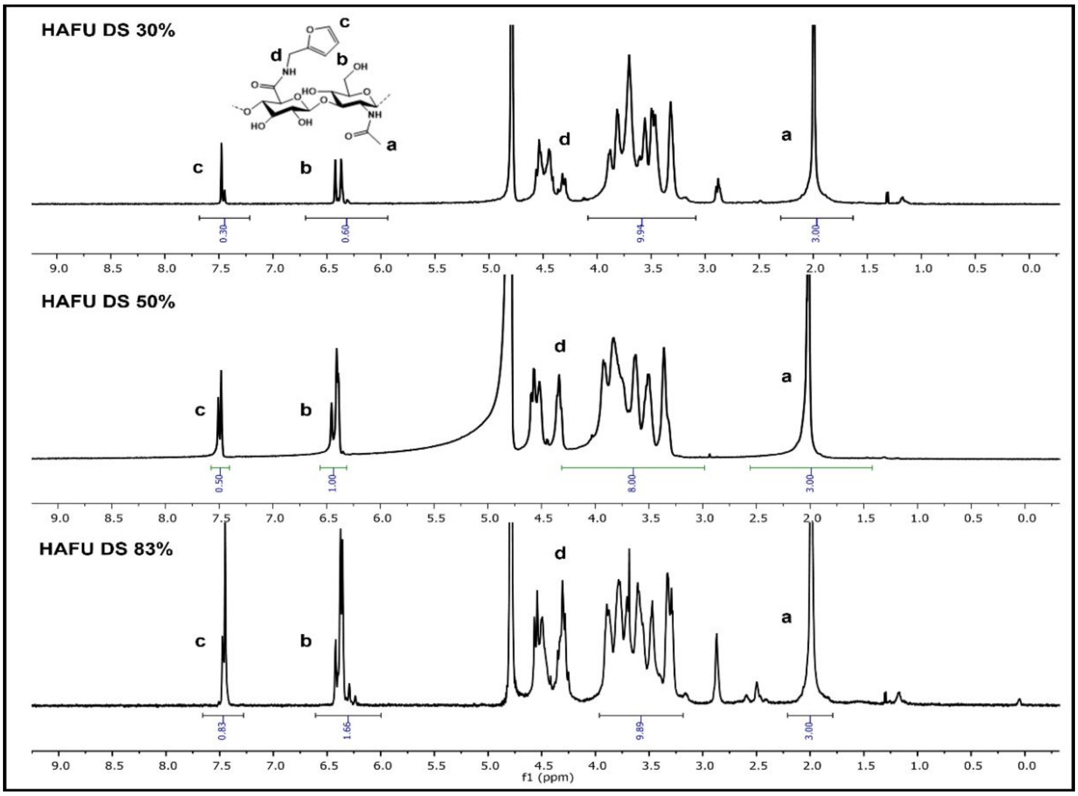

Figure S1. ^{1}*H*-NMR spectra of HAFU DS ~ 30, 50, 83% . D_2O was used as solvent.

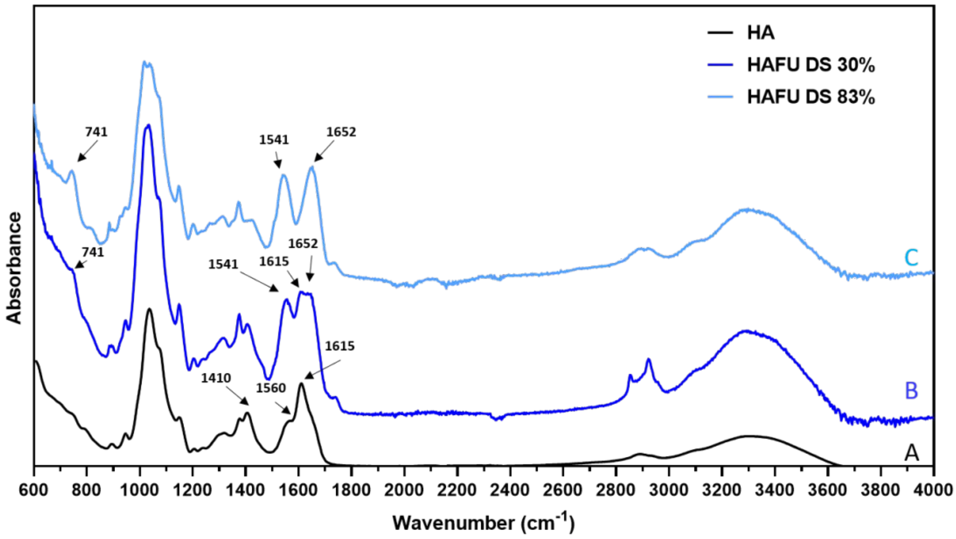

Figure S2. FTIR spectra of A) HA, B) HA-FU DS 30% and C) HAFU 83%. Unmodified HA has spectral bands at 1615 and 1410 cm^{-1}, assigned to the asymmetric and symmetric stretching vibration of the carboxylate anion, respectively. HA spectra are also characterized by an amide II bond at 1560 cm^{-1}. The grafting of furan groups to HA (HAFU) after the reaction of HA with furfurylamine is indicated by shift and increase of the peaks at 741, 1541 and 1652 cm^{-1} from the HAFU DS 30% and even more for HAFU DS 83% derivative corresponding to the furan moieties. The functionalization of HA with furan groups is also demonstrated by the decrease in the absorbance of carboxylate anion (1410 cm^{-1}) peak for the HAFU DS 30% and even more for DS 83%.

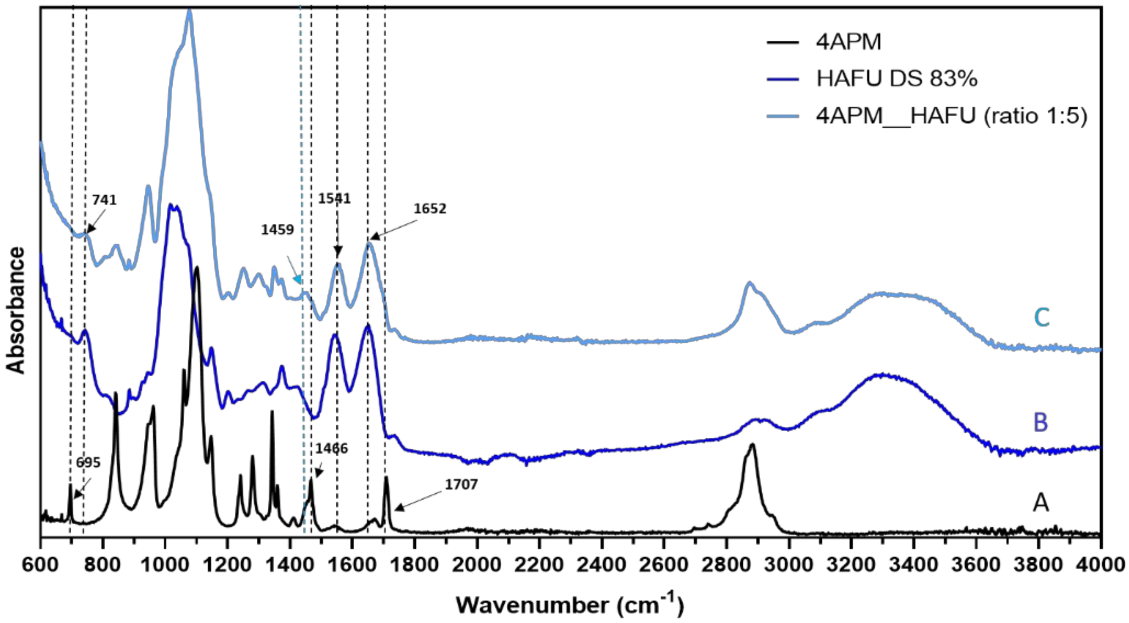

Figure S3. FTIR spectra of: A) 4APM with arrows showing the C=C absorbance at 1466 cm^{-1}, -C=O stretch vibration at 1707 cm^{-1} and the =C-H- bending vibration at 695 cm^{-1} from

maleimide. B) HAFU DS 83% with arrows indicating bonds of amide I stretching, amide II bending at 1652, 1541 cm^{-1} respectively, and =C-H- bend of furan moiety 741 cm^{-1}. C) The cross-linked HAFU-4APM hydrogel (molar ratio maleimide: furan 1:5) showing the absence of the 695, 1466 and 1707 cm^{-1} maleimide peaks, decreased 741, 1541 cm^{-1} furan peak and the appearance of a new peak at 1459 cm^{-1} corresponding to the C=C bond in the Diels-Alder adduct.

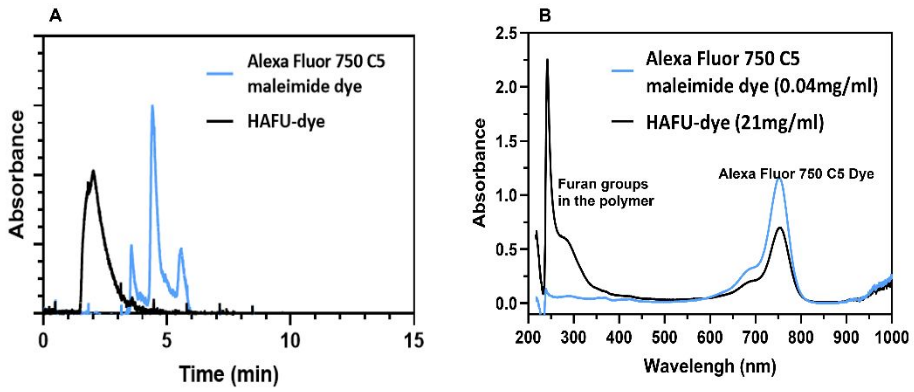

Figure S4. A) SEC chromatograms of HAFU-750dye polymer in black and Alexa fluor 750 C5 maleimide dye in blue. B) UV-vis spectra of HAFU-750dye polymer in black and Alexa fluor 750 C5 maleimide dye in blue.

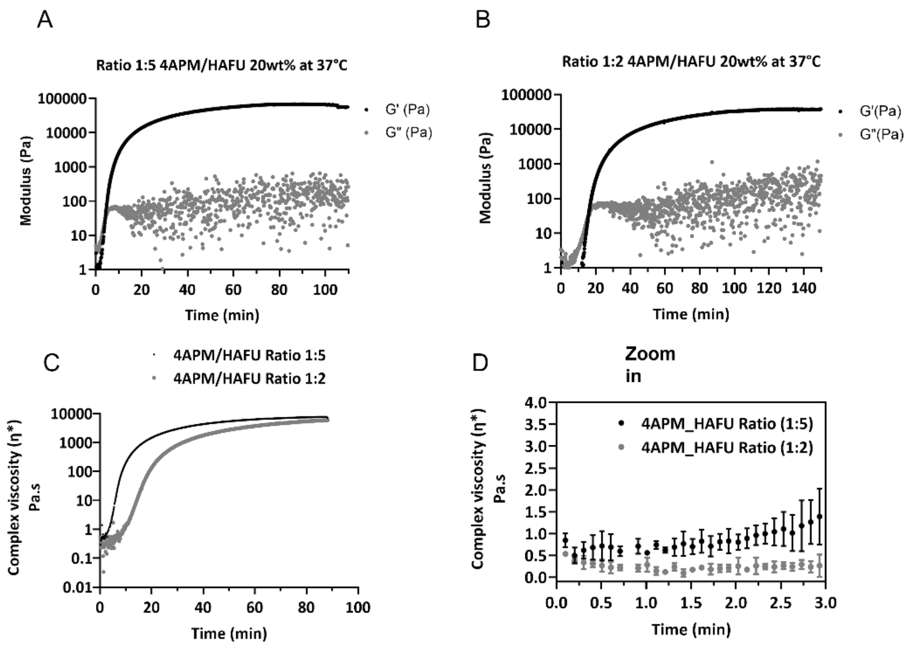

Figure S5. Rheological and mechanical characterization of 4APM-HAFU hydrogel formulations. Evaluation of storage (G') and loss modulus (G'') as a function of time in 20 wt% 4APM-HAFU hydrogel, A) molar ratio of maleimide/furan 1:2 and B) molar ratio 1:5. C) Complex viscosity (η*) of 20 wt% 4APM-HAFU hydrogel at 37 °C. D) Complex viscosity (η*) of 20wt% 4APM-HAFU hydrogel at 37°C time (0-3 minutes).

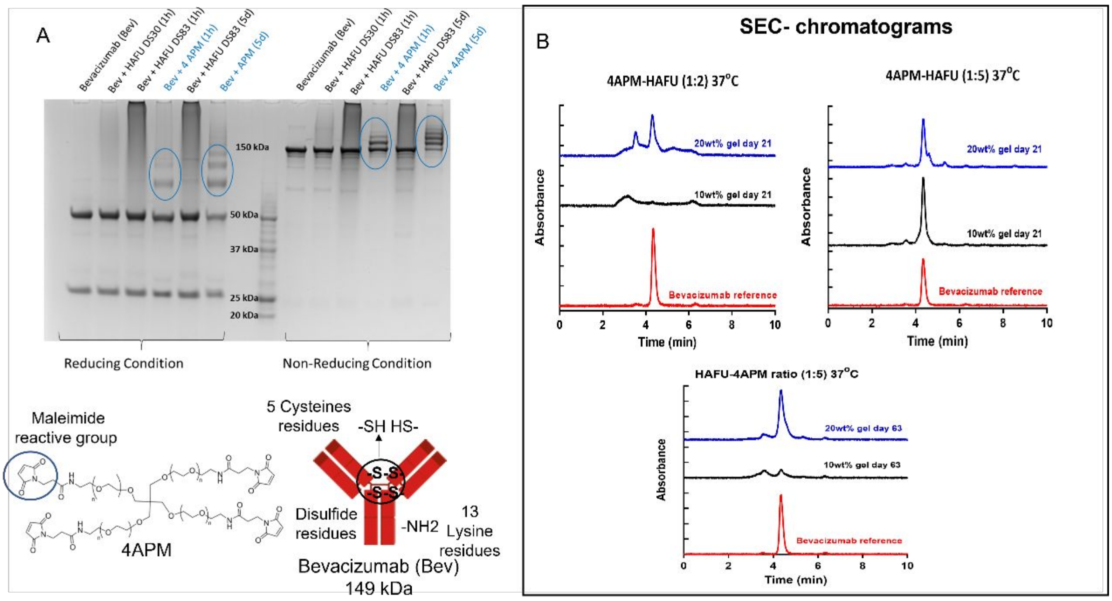

Figure S6. A) SDS-page of bevacizumab after incubation with hydrogel precursors at 37 °C for 1 hour and 5 days. The maleimide present in the 4APM crosslinker conjugated to the proteins, and therefore, its molecular weight was increased. B) SEC chromatograms of 10-20 wt% 4APM-HAFU hydrogels at 1:2 and 1:5 ratio after 21-63 days of release.

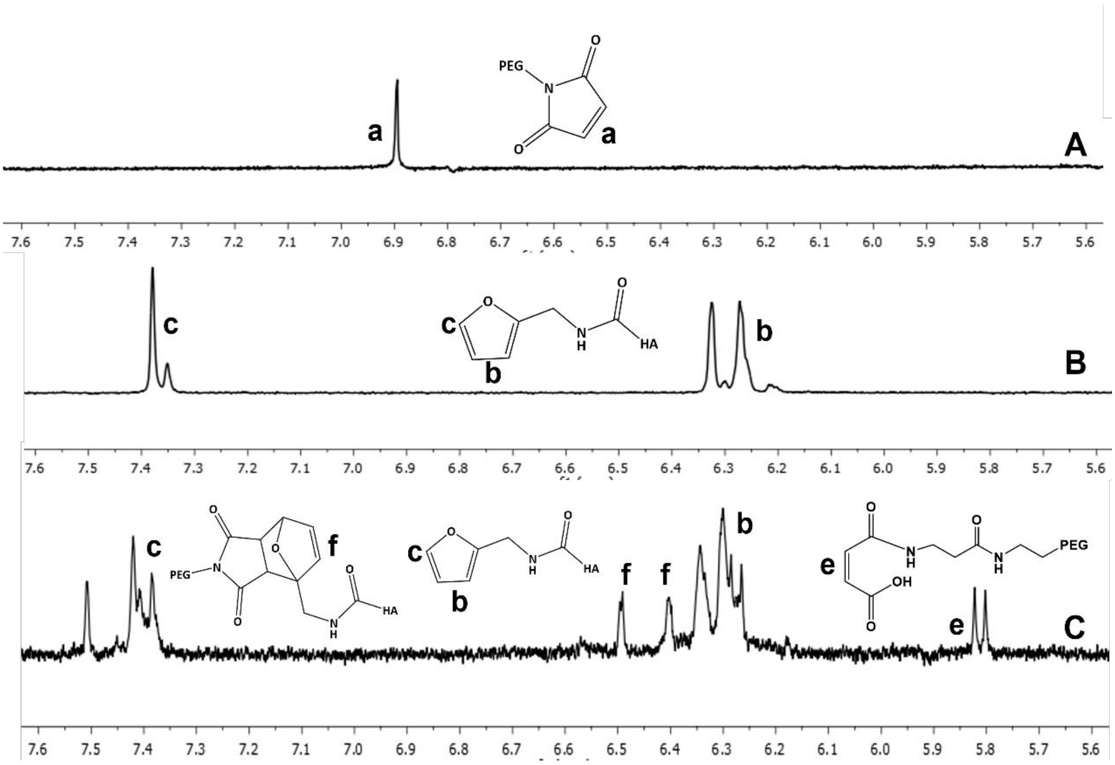

Figure S7: ^{1}H-NMR spectra in D_20 (between 5.6-7.6 ppm) of A) 4APM crosslinker, indicating the presence of maleimide protons (a). B) HAFU polymer, indicating the presence of furan protons (b and c). C) Freeze-dried degradation product obtained during swelling and degradation studies upon the complete dissolution of hydrogel network in aqueous medium. Protons of furan (c and b) and the Diels-Alder adduct (f)

are found. No intact maleimide protons are found (a), while the protons of the hydrolyzed maleimide product were identified (e).

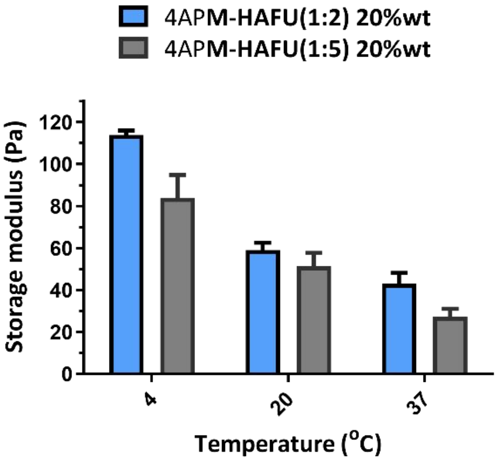

Figure S8 Storage modulus (Pa) at the gelation point (here defined as the time at which G’ equals G”) of 20wt% 4APM-HAFU hydrogel formulations at molar ratio 1:2 and 1:5 maleimide: furan as a function temperature (37, 20, and 4 °C).

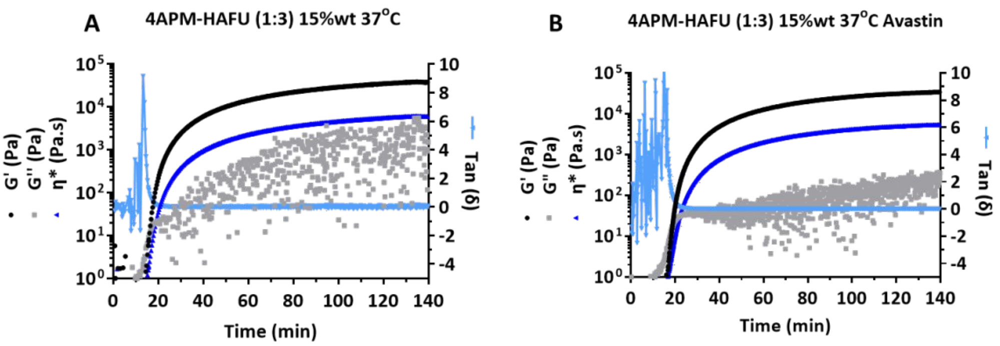

Figure S9: Storage (G’), loss modulus (G”) and tan(δ) as a function of time of 15wt% 4APM-HAFU (molar ratio 1:3) hydrogel formulation with and without bevacizumab

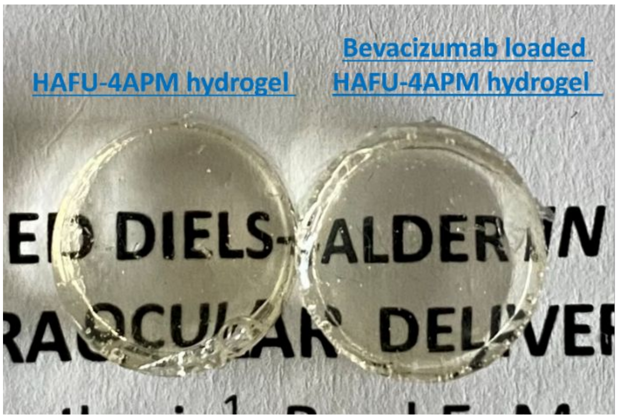

Figure S10: Image of transparent 20 wt% HAFU-4APM hydrogels (diameter 8 mm, 4 mm height) formed at 37 °C with and without loading of bevacizumab.
